# Co-designing an interprofessional care pathway for (risk of) malnutrition and sarcopenia in community-dwelling older adults

**DOI:** 10.1186/s12913-026-14047-7

**Published:** 2026-01-20

**Authors:** Sandra D. Boxum, Jan-Jaap Reinders, Manon G. A. van den Berg, Job van’t Veer, Michael Tieland, Sophie L. W. Spoorenberg, Anjo Geluk-Bleumink, Philip J. van der Wees, Hans Drenth, Harriët Jager-Wittenaar

**Affiliations:** 1 Research Group Healthy Ageing, Allied Health Care and Nursing, Hanze University of Applied Sciences, Groningen, the Netherlands; 2 Department of Gastroenterology and Hepatology, Dietetics Radboud university medical center, Nijmegen, the Netherlands; 3 Center for Dentistry and Dental Hygiene, University Medical Center Groningen, University of Groningen, Groningen, the Netherlands; 4 Research Group Interprofessional Education (IPE), Lifelong Learning, Education and Assessment Research Network (LEARN), Research Institute SHARE, University Medical Center Groningen University of Groningen, Groningen, the Netherlands; 5 Researchgroup Digital Innovation in Healthcare, Department of Healthcare, NHL Stenden University of Applied Sciences, Leeuwarden, The Netherlands; 6 Center of Expertise Urban Vitality, Faculty of Sports and Nutrition, Amsterdam University of Applied Sciences, Amsterdam, the Netherlands; 7 School of Exercise and Nutrition Sciences, Institute for Physical Activity and Nutrition, Deakin University, Geelong, Victoria, Australia; 8 Primary care group ‘Dokter Drenthe’, Assen, the Netherlands; 9 Denktank 60+ Noord, Steenwijk, the Netherlands; 10 Science Department IQ Health, Radboud university medical center, Nijmegen, the Netherlands; 11 Department of Rehabilitation, Radboud university medical center, Nijmegen, the Netherlands; 12 Organization for Elderly Care ZuidOostZorg, Drachten, the Netherlands; 13 Department of Primary and Long-Term Care, Cure and Care in the Community Context (FOUR-C) research program, University of Groningen, University Medical Center Groningen, Groningen, the Netherlands; 14 FAITH research, Groningen & Leeuwarden, Leeuwarden, the Netherlands; 15 Department of Physiotherapy, Human Physiology and Anatomy, Faculty of Physical Education and Physiotherapy, Research Unit Experimental Anatomy, Vrije Universiteit Brussel, Brussels, Belgium

**Keywords:** Care pathway, Interprofessional collaboration, Malnutrition, Sarcopenia, Design-oriented approach

## Abstract

**Background:**

Integrating care for (risk of) malnutrition and sarcopenia in primary care is challenging, as limited physical proximity among healthcare professionals hinders collaboration. Both health conditions are common in community-dwelling older adults and are associated with significant declines in physical functioning, independence, and quality of life. Healthcare professionals tend to manage malnutrition and sarcopenia separately, leading to missed opportunities for early (risk) identification and coordinated care. An interprofessional care pathway can provide an evidence-based, structured framework to support such integration. Therefore, we aimed to co-design an interprofessional care pathway for addressing (risk of) malnutrition or sarcopenia in community-dwelling older adults within the Dutch primary care context.

**Methods:**

We applied a design-oriented approach using the Double Diamond model to guide the development process across the discover, define, and develop phases. Methods included persona development and validation, desk research, patient journey mapping, service blueprinting, and prototyping. In addition, we introduced an interprofessional visualisation combining elements of the patient journey map and service blueprint to represent both front-stage and back-stage care processes along a timeline. The data were analysed iteratively.

**Results:**

Thirteen healthcare professionals, including district nurses, dietitians, physiotherapists, general practice assistants, dementia case managers, general practitioners, and a geriatric specialist, participated in the co-design process. The process comprised three in-person sessions and two online follow-up meetings. In addition, one community-dwelling older adult was interviewed. The co-design process resulted in a prototype interprofessional care pathway that offers a structured workflow for detecting, screening, and managing (risk of) malnutrition and sarcopenia. The pathway addresses interprofessional, person-centred, and integrated care by a designated point of contact, shared treatment plans, continuous interprofessional communication, shared decision-making, clearly defined roles, and regular team evaluation. The pathway includes practical tools such as detection cards, templates and formats for task allocation, work agreements, and team evaluation.

**Conclusion:**

This study presents a co-designed prototype of an interprofessional care pathway to address (risk of) malnutrition and sarcopenia in community-dwelling older adults. Future research should evaluate its feasibility in daily primary care practice.

**Supplementary Information:**

The online version contains supplementary material available at 10.1186/s12913-026-14047-7.

## Background

The integration of care for complex conditions such as (risk of) malnutrition and sarcopenia presents challenges within the primary care system [1, 2], where limited physical proximity among healthcare professionals (HCPs) often hinders collaboration [3–5]. Malnutrition and sarcopenia are prevalent among community-dwelling older adults [6, 7] and are associated with substantial declines in physical functioning, independence, quality of life, and survival [8–12]. HCPs often manage malnutrition and sarcopenia independently rather than in collaboration [2], resulting in missed opportunities for early (risk) identification, timely intervention, and coordinated follow-up [13, 14].

One promising strategy to address these shortcomings is to improve care integration through workflow standardisation [15]. This can be operationalised via an interprofessional care pathway, which offers a structured framework to support coherent, evidence-based, and well-coordinated care delivery [16, 17]. However, to date, no interprofessional care pathway has yet been developed to address (risk of) malnutrition and sarcopenia in community-dwelling older adults.

Such a pathway must build upon evidence-based interventions that target both the causes and manifestations of malnutrition and sarcopenia, which are distinct yet interrelated conditions. Malnutrition is an acute or chronic condition resulting from insufficient nutrient intake or absorption, leading to adverse changes in body composition [18]. Sarcopenia is a muscle disease marked by reduced muscle mass and strength, commonly arising from physical inactivity and poor nutritional intake [19]. While the combination of loss of both muscle mass and muscle strength characterises sarcopenia, low muscle mass alone is a characteristic of malnutrition [18]. Malnutrition increases the risk of sarcopenia [20], and inadequate protein–energy intake together with functional limitations can sustain a bidirectional cycle linking both conditions [21]. Management of malnutrition and sarcopenia requires integrated nutritional and exercise interventions, which improve physical performance and nutritional status in older adults at risk of, or living with, these conditions [22, 23].

Integrating these interventions requires interprofessional collaboration (IPC), as no single HCP possesses all the necessary expertise to assess, treat, and monitor (risk of) malnutrition and sarcopenia. IPC occurs when HCPs within a shared problem domain, such as (risk of) malnutrition and sarcopenia, engage interactively to act or decide on relevant care issues [24]. It is characterised by shared accountability and clearly defined roles and goals [25, 26]. Evidence shows that IPC enhances timely intervention, treatment adherence, and patient outcomes, while significantly reducing emergency waiting times, inpatient stays, and redundancies in care delivery [15, 27–30].

Care pathways are effective in improving clinical outcomes, but do not automatically enhance IPC [31]. Care pathways primarily support process coherence by standardising procedures, clarifying roles, and enabling shared decision-making among HCPs [15–17]. Coherence in IPC can be conceptualised along three interconnected dimensions: (1) social coherence, which refers to strong, respectful relationships among professionals [32, 33]; (2) content coherence, which involves integrating diverse expertise [34]; and (3) process coherence, which relates to the alignment of workflows and structural enablers [35, 36]. A care pathway supports interprofessional teamwork through coordination and communication, but does not fully address relational coordination [37]. Thus, interprofessional care pathways are one essential component for strengthening collaboration.

Co-design, a participatory approach with diverse stakeholders, can foster shared ownership, enhance contextual relevance, and promote commitment to future implementation of an interprofessional care pathway [38, 39]. It can make front-stage patient-facing interactions and back-stage coordination between professionals in IPC explicit [40]. Differences between professional groups arise from distinct frames of reference and discipline-specific priorities. Because professionals act on what they notice and value in their roles (professional identity triggers), their reasoning and actions vary across contexts, shaping how they express their professional identity [41]. The exact mechanism by which interprofessional identity is likely activated when cues (interprofessional identity triggers) clearly relate to the shared context and problem [41, 42]. These differences do not imply that any profession’s perspective is superior. Given that each professional is competent, those differences can be considered complementary within their professional standards. Accordingly, co-design is well-suited to engaging non-designers and uses accessible methods and short iterative cycles to bring these complementary perspectives together [43]. It supports early, meaningful involvement of diverse HCPs, including general practitioners, practice assistants, dietitians, physiotherapists, and nurses.

Accordingly, we aimed to co-design an interprofessional care pathway for (risk of) malnutrition or sarcopenia in community-dwelling older adults within the Dutch primary care context.

## Methods

### Study design

**Fig. 1:**
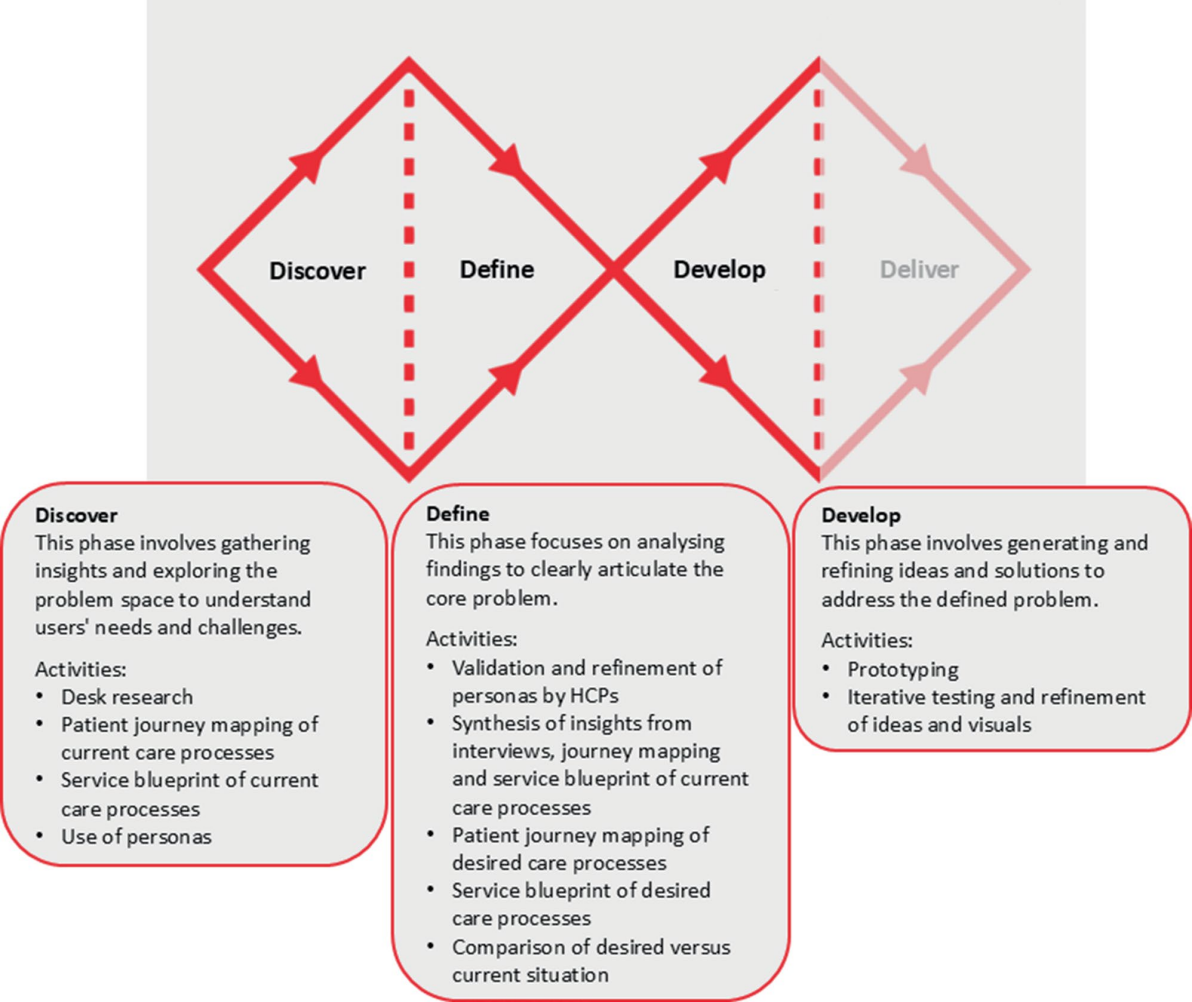
Structuring of research activities across the double diamond model [44]

We used a design-oriented approach based on the Double Diamond model [Fig. 1]^.^ [44]. The Double Diamond model is recognised for guiding individuals in designing solutions to complex problems. The model is organised into two “diamonds”, reflecting a structured process alternating between divergent and convergent thinking. It consists of four phases: discover, define, develop, and deliver. Its iterative character allows flexibility in revisiting earlier phases as new insights emerge. This study focused on the discover, define, and develop phases. We operationalised this in a multi-stakeholder context using co-design, defined as a participatory, reflexive and creative decision-making process that draws on diverse stakeholder knowledge to identify challenges and co-develop solutions in complex adaptive systems [45].

To create optimal conditions for interprofessional co-design, we applied an evidence-informed methodological approach. We collected baseline characteristics to contextualise how professional background, interprofessional competence and interprofessional identity might shape contributions (see *Baseline characteristics*). Interprofessional identity may be influenced by professional self-efficacy and motivational beliefs [46], and interprofessional identity triggers can promote speaking up in mixed-profession settings [42]. Although not empirically tested here, these considerations informed the design of the co-design set-up, with mixed-profession groups, explicit role-clarity prompts, and shared artefacts to support meaningful interprofessional co-design.

The Consolidated Criteria for Reporting Qualitative Research (COREQ) checklist was used to ensure accurate reporting of the study’s design, conduct, analysis, and findings due to the qualitative nature of the primary data [47].

### Study setting

This study was conducted within the context of Dutch primary care. In the Netherlands, older adults who live independently receive care and support at home through the Social Support Act 2015 (Wet Maatschappelijke Ondersteuning, WMO 2015) via municipalities, as well as through the Health Insurance Act (Zorgverzekeringswet, Zvw). The Dutch healthcare system provides accessible primary care, with general practitioners (GPs) as gatekeepers. GP care is generally covered by basic health insurance, although some services may require additional coverage. Other primary healthcare services, such as dietetics and physiotherapy, are either only partially reimbursed or, in specific cases, reimbursed under basic health insurance (Zvw) or require supplementary insurance [48].

### Study population

A purposive recruitment strategy was employed to engage HCPs from diverse professional backgrounds and to recruit older adults who had experience receiving care from multiple professionals and who affirmed that they could recall their patient journeys. HCPs and older adults were recruited by contacting individuals who had previously consented to future study participation during a prior interview study [49, 50]. A call for participation was also disseminated through professional networks, which targeted general practitioners (GPs), GP assistants/nurses, case managers, physiotherapists, dietitians, and district nurses in the northern provinces of the Netherlands. Interested potential participants contacted the first researcher via email or phone.

HCPs working in primary care with community-dwelling older adults diagnosed with or at risk of malnutrition or sarcopenia in the Netherlands were eligible to participate. Eligible older adults were aged ≥65 years, community-dwelling, under the care of a GP, with (risk of) malnutrition or sarcopenia, and receiving or having received treatment related to these health conditions.

Stratification ensured a balanced representation of professional backgrounds, aiming to include at least one representative from each professional background in every session. Additional recruitment was conducted when needed to broaden the perspectives in the co-design process. To explore the alignment between professional perspectives and those of older adults, we planned to include up to four older adults, depending on the richness and complementarity of the data.

### Data collection procedures

Data were collected through one-on-one interviews with older adults and multiple in-person and online co-design sessions with HCPs between February and July 2024. Fig. 1 illustrates how the Double Diamond model guided the co-design process, outlining the research activities undertaken in each phase. Older adults were engaged at the Consult level (i.e., obtain feedback on analyses and alternatives and show how input influenced decisions) as defined in the International Association for Public Participation (IAP2) Spectrum during the co-design process [51]. HCPs participated at the Collaborate level (i.e., partner across stages to co-develop options and identify the preferred solution).

HCPs participated in one or more co-design sessions based on availability and interest. Co-design sessions were considered complete when no new insights or perspectives emerged from the discussions. Older adults participated in individual interviews rather than group settings or co-design sessions with HCPs. This decision was based on our previous research, which identified challenges to focus group participation, including reluctance to participate in group discussions and a preference for one-on-one interviews [50]. Furthermore, the co-design sessions primarily addressed the back-stage processes of IPC, which are relevant to HCPs. Older adults, on the other hand, are more directly involved in front-stage processes, such as direct care delivery and their interactions with HCPs. For this reason, individual interviews were deemed more appropriate for their participation [44].

The interviews and discussions were audio-taped, and the recordings were deleted after the first author (SB) took written notes. SB moderated the co-design sessions, with JJR as co-moderator. SB is a PhD candidate with a background in physiotherapy. SB was trained and experienced in conducting focus groups and interviews. JJR is a postdoctoral researcher with a background in work and organisational psychology. HJW and HD participated in co-design sessions, contributing to their expertise on malnutrition and sarcopenia. Participants were informed about the researchers’ roles and the study objectives before the co-design sessions. Additionally, sessions were held with the research team members (SB, JJR, MvdB, MT, HD, PvdW, HJW) to discuss the session notes and findings from the co-design sessions, with different team members participating at various stages and times. The team comprised individuals with diverse expertise, including nutrition and physical activity (MT), nutrition and dietetics (MvdB, HJW), physiotherapy (SB, HD, PvdW), gerontology (HD), and work and organisational psychology (JJR). Each member interpreted and contributed inputs to the design sessions and the care pathway from their respective areas of expertise.

### Baseline characteristics

Baseline characteristics included the professional backgrounds of the HCPs and the results of two validated questionnaires administered during the in-person co-design sessions: the Extended Professional Identity Scale (EPIS) [52] and the Chiba Interprofessional Competency Scale (CICS29) [53]. The EPIS assesses an individual’s interprofessional identity. It consists of three subscales: interprofessional belonging, interprofessional commitment, and interprofessional beliefs. Higher scores on the EPIS indicate a stronger sense of interprofessional identity, characterised by a more significant internalised motivation towards IPC [52]. The CICS29 evaluates a professional’s ability to collaborate effectively with colleagues from different disciplines [53]. It includes six subscales: attitudes and beliefs as a professional, team management skills, actions to achieve team goals, providing care with respect for client/patient preferences, attitudes and behaviour that enhance team cohesion, and fulfilling your role as a professional. Higher scores on the CICS29 reflect greater competence and openness towards IPC.

### Data collection methods

First, the current care delivery for (risk of) malnutrition and sarcopenia was explored, followed by the desired situation. Desk research, personas, patient journey maps, service blueprints, and prototyping were used to make context and perceptions explicit, eliciting what each profession notices first (professional identity triggers) and what prompts joint action (interprofessional triggers).

### Desk research

Desk research was conducted in two parts. First, we drew on findings from our earlier qualitative research, including interviews and focus groups on needs, preferences, and perceived barriers and facilitators to IPC from professionals’ and older adults’ perspectives [49, 50]. Insights from this earlier work were synthesised and used as input for the co-design sessions.

Second, we explored existing international and national protocols and guidelines related to the care of older adults with (risk of) malnutrition and sarcopenia, as well as relevant literature on IPC. Although the Dutch guidelines are based on international evidence and guidelines, they offer more context-specific and practice-oriented guidance suited to implementation in the Dutch primary care setting [54]. The insights gained from the desk research informed the development of targeted questions and helped guide discussions during the co-design sessions. In addition, the findings supported the alignment of the care pathway with current care standards and protocols.

### Persona development and validation

Personas were developed based on interviews and focus groups with older adults conducted in our previous research [50]. These personas represent fictional but realistic profiles of older adults with malnutrition or sarcopenia [55, 56]. Personas provide detailed, realistic representations of the target population, enabling HCPs to better understand the needs and challenges older adults face throughout their care journey. To ensure accuracy, HCPs assessed the relevance, completeness, and realism of the content during co-design sessions. Based on their feedback, adjustments were made, and the refined personas were presented in a follow-up session for final approval.

### Patient journey mapping

An interprofessional patient journey map was created to visualise the front-stage experience of older adults interacting with healthcare services for malnutrition and sarcopenia care [40]. This adapted version of a traditional journey map accounts for multiple professional perspectives, capturing various care paths that converge around the same client [Additional file 1]. During patient-journey mapping, the priority was on eliciting what each profession notices first and where they would act (professional identity). The journey mapping process was conducted in two ways:

1. First, during an interview, the researcher and the older adult constructed a patient journey map. This interview was structured around the patient journey mapping method and did not follow a fixed set of questions, therefore, no formal interview guide was developed or used.
2. Subsequently, HCPs constructed a journey map in subgroups based on the different personas representing an older adult with malnutrition or sarcopenia. HCPs imagined how an older adult might navigate the patient journey, empathising with the challenges they could face as care recipients. In addition to mapping the current situation, HCPs were also asked to visualise a desired future journey, outlining an optimal care process from a patient’s perspective.

Outputs from both the older adult interview and the HCP exercises were compared to identify alignments and discrepancies, ensuring concordance with real patient experiences and revealing potential gaps in professional assumptions [40, 56]. Differences and process gaps were subsequently fed back to HCPs during co-design sessions to inform improvements to the care pathway.

### Service blueprint development

To expand upon the patient journey maps, a service blueprint was created to visualise the care process, including both front-stage (patient-facing) and back-stage (professional/supporting) activities [40, 56]. The priority in conducting the service blueprint was to surface signals that enable HCPs to recognise when collaboration with other professions is needed and to activate interprofessional identity, prompting joint action. The blueprint mapped patient–HCP contact points horizontally (the patient journey) and interprofessional back-stage interactions vertically on a timeline [Additional file 1]. Subsequently, a second service blueprint was created to explore potential improvements and design a desired future care process that aligns with patient needs and interprofessional practice.

### Prototyping

The research team synthesised insights from desk research, patient journey mapping, and service blueprinting into a prototype of an interprofessional care pathway for (risk of) malnutrition and sarcopenia. Components of the care pathway were presented to HCPs during online sessions to refine this prototype. These sessions focused on the clarity, relevance, and usability of the pathway in daily practice. The feedback gathered during these discussions informed iterative refinements, ensuring that the final version of the care pathway aligned with user needs and was feasible within professional routines. Additionally, we obtained feedback on the detection card for older adults and their family and/or network. An older-adult representative circulated the Dutch version among older adults and collected structured feedback on first impression, look and feel, clarity, missing information, unclear wording, and other suggestions.

## Data analysis

Quantitative baseline data from the EPIS and CICS29 questionnaires were summarised descriptively in Microsoft Excel (version 2504). For each subscale, participant-level scores were calculated as the mean of the constituent items (yielding a 1–5 Likert metric). We reported the median (IQR; P25–P75) and the mean (range; min–max) across participants.

The analysis of the co-design sessions followed an iterative, co-creative process in which researchers and HCPs were actively involved. Findings from previous sessions were presented at the start of each co-design session, enabling HCPs to reflect, discuss, and refine insights. The iterative nature of the analysis enabled ongoing refinement. Each session was built on the findings of previous sessions, allowing continuous adaptation and validation by HCPs.

## Results

Thirteen HCPs and one older adult participated in this study. The research team members involved in data collection (SB, JJR, HD, and HJW) had no prior personal relationships with any of the participants. Despite recruitment efforts, only one older adult, an 87-year-old woman, consented to participate in a one-on-one, 60-minute interview session at home. Another older adult showed interest but ultimately did not respond. Reported reasons for non-participation included health concerns and pre-existing commitments within an already demanding schedule.

The co-design process with HCPs consisted of three 2.5-hour in-person sessions and two 1.5-hour online follow-up sessions via Microsoft Teams (version 24,102.22142869.7475). Ten HCPs participated in at least one in-person co-design session and completed the EPIS and CICS29 questionnaires. During the online follow-up sessions, three additional HCPs who had not joined the in-person sessions participated. In total, 13 HCPs contributed to the co-design process. Three HCPs who had initially planned to participate in a session had to cancel due to illness. Table 1 presents the professional backgrounds of participating HCPs for each session.

**Table 1:** Professional background of HCPs per session

Professional background	Co-design 1	Co-design 2	Co-design 3	Online 1	Online 2
District nurse	1	1	1	-	-
Dietitian	1	2	1	3	2
Physiotherapist	1	1	-	1	2
General practice assistant/nurse	1	2	1	2	1
Dementia case manager	1	1	1	1	-
General practitioner	-	1	1	1	-
Geriatric specialist	-	-	-	1	-
Combination role*	1	1	-	-	1
**Total number of participants**	**6**	**9**	**5**	**9**	**6**

* *Combination role of general practice nurse, dementia case manager, and district nurse*

Table 2 and 3 present baseline EPIS and CICS-29 results. Median subscale scores were generally toward the upper end of the 1–5 scale, suggesting relatively strong interprofessional identity and competence (*n* = 10). Table 4 outlines the key activities per session with HCPs.

**Table 2:** Extended professional identity scale (EPIS; *n* = 10)

Subscale	Median (IQR)	Mean (range)
1.Interprofessional belonging	4.3 (4.1–4.7)	4.4 (4.0–5.0)
2.Interprofessional commitment	4.3 (4.0–4.3)	4.2 (3.5–5.0)
3.Interprofessional beliefs	4.2 (4.0–4.5)	4.1 (2.0–5.0)

*Note: Subscale scores are participant-level means of multiple Likert items; responses were measured on a 5-point Likert scale: 1 = strongly disagree, 2 = disagree, 3 = neutral, 4 = agree, 5 = strongly agree. IQR = interquartile range (P25–P75)*

**Table 3:** Chiba interprofessional competency scale (CICS29; *n* = 10)

Subscale	Median (IQR)	Mean (range)
1.Attitudes and beliefs as a professional	4.3 (4.2–4.5)	4.3 (4.0–4.8)
2.Team management skills	3.8 (3.6–4.0)	3.8 (2.8–4.8)
3.Actions to achieve team goals	4.0 (3.9–4.2)	4.0 (3.6–4.4)
4.Providing care with respect for client/patient preferences	4.6 (4.2–5.0)	4.5(4.2–5.0)
5.Attitudes and behaviour that enhance team cohesion	3.9 (3.6–4.0)	3.8 (3.0–4.5)
6.Fulfilling your role as a professional	4.2 (4.0–4.4)	4.2 (3.5 -4.8)

*Note: Subscale scores are participant-level means of multiple Likert items; responses were measured on a 5-point Likert scale: 1 = No, 2 = Not so much, 3 = Neither Y/N, 4 = Fairly well, 5 = Yes. IQR = interquartile range (P25–P75)*

**Table 4:** Key activities per session

Session	Key activities
In-person session 1	Persona validation; current patient journey mapping (12-month timeline); current use of screening tools (malnutrition/sarcopenia); treatment start criteria; funding and collaboration arrangements; guideline use.
In-person session 2	Desired patient journey mapping (12-month timeline); preferred use of screening tools (malnutrition/sarcopenia); treatment start criteria; funding and collaboration arrangements; guideline use; service blueprint (roles, front-/back-stage actions, touchpoints, support processes).
In-person session 3	Completeness check of the desired 12-month pathway; 3-month “zoom-in”; define roles and tasks by profession; outline pathway steps.
Online follow-up 1	Prototyping and refining the pathway; clarifying the aim, target users, and content; focused discussion on detection, screening, and diagnosis; targeted questions on the prototype’s clarity, structure, and accessibility.
Online follow-up 2	Final prototyping; review two overview pages (preconditions for interprofessional collaboration; visual flow for detection–screening–treatment); discuss guideline use and barriers (malnutrition/sarcopenia); agree preferred channels/formats for the care pathway.

## Exploring the current care delivery

### Desk research

Findings from our previous research highlighted preferences, needs, facilitators, and barriers experienced by professionals in IPC and the wishes and needs of community-dwelling older adults with (risk of) malnutrition and sarcopenia [49, 50]. Professionals emphasised the importance of smooth information exchange, clear role distribution, and care coordination around the older adult. They also expressed the need for informal caregivers, social workers, and insurers to be involved. Older adults, in turn, called for active participation in their treatment, well-informed professionals, and effective collaboration across disciplines.

### Personas

Additional File 2 presents the four initial personas, and Additional File 3 presents the four validated personas from the second co-design session.

### Patient journey map

A patient journey map was developed based on a structured, open-ended interview with an older adult who had experienced malnutrition and sarcopenia. The 60-minute in-home interview provided insights into her healthcare journey and interactions with various HCPs, such as the GP, GP assistant, district nurse, dietitian, and physiotherapist. While initially sceptical about the need for home nursing care, the older adult later appreciated the support. She also appreciated the straightforward access to the GPs assistant and the ability to manage her nutritional supplements. However, gaps in follow-up care were evident, such as the failure to restart physiotherapy after other health issues arose. At certain moments, she was surrounded by multiple HCPs, yet overall, the journey map indicated that care was delivered in a fragmented manner. These findings aligned with the interprofessional patient journey maps created by HCPs during the co-design sessions, based on the personas.

### Service blueprint

**Fig. 2:**
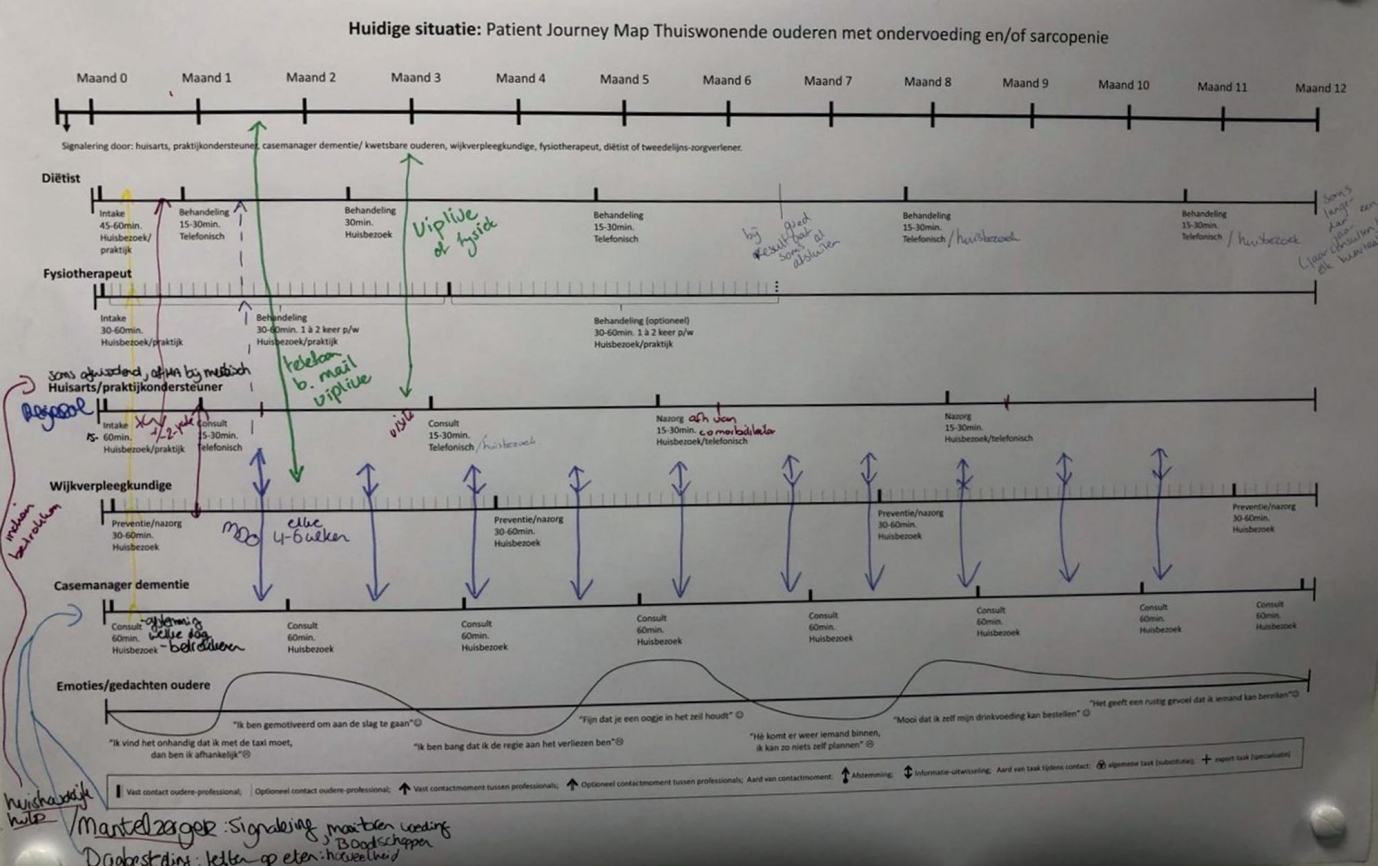
Interprofessional patient journey and service blueprint integrating front-stage (horizontal) and back-stage care processes (vertical). Legend: the figure shows an example of a 12-month timeline with parallel care lanes for the various HCPs, i.e., dietitian, physiotherapist, GP/GP assistant, district nurse, dementia case manager, and emotions/thoughts of the older adult. Tick marks along the horizontal lanes indicate the type of contact between the professional and the community-dwelling older adult (consultation, home visit, telephone). Vertical arrows denote interactions between HCPs. The lower curve traces the older adult’s emotions and concerns across the journey. Abbreviations: GP, general practitioner; HCPs, healthcare professionals

During the first and second co-design sessions, HCPs created service blueprints for the current care delivery process for older adults with (risk of) malnutrition and sarcopenia (Fig. 2). The blueprint revealed significant gaps in communication and coordination between HCPs, mainly between allied HCPs (such as dietitians and physiotherapists) and other care providers. Additionally, the service maps revealed duplicated efforts, as initial assessments were often uncoordinated across disciplines, resulting in overlapping patient histories being collected during the first consultations. Multiple home visits occurring within the same week led to inefficiencies and increased the burden on older adults.

However, promising examples of interprofessional information exchange were mentioned. For instance, a district nurse weighing a patient and taking the initiative to pass that information along to the GPs assistant or dietitian for monitoring and to support further treatment.

HCPs mentioned that multidisciplinary meetings were typically scheduled at fixed intervals, such as once every four to six weeks. However, allied HCPs were often not included in these meetings. Moreover, the discussions during these meetings focused primarily on individual patients with complex care needs, with limited attention given to broader collaboration strategies or shared care planning across disciplines.

## Exploring the desired care delivery

### Desk research

Several international and national guidelines were deemed appropriate to inform diagnostics and management for (risk of) malnutrition and sarcopenia in the care pathway.

The international guidelines included the Global Leadership Initiative on Malnutrition (GLIM) and the European Working Group on Sarcopenia in Older People (EWGSOP2) criteria [18, 19], as well as the European Society for Clinical Nutrition and Metabolism (ESPEN) guideline on clinical nutrition and hydration in geriatrics, and the World Health Organisation (WHO) guideline on physical activity and sedentary behaviour [57, 58].

National guidelines included the Allied Healthcare Guideline for Frail Older Adults (Paramedische richtlijn voor kwetsbare ouderen), the Guideline for Malnutrition (Richtlijn ondervoeding), The Wheel of Five from the Dutch Health Council (Schijf van vijf van de Gezondheidsraad), and the KNGF Standard Exercise Intervention for Frail Older Adults (KNGF standaard voor kwetsbare ouderen) [59–62]. In addition, the Belgian Sarcopenia Guideline was considered relevant [63].

We also explored the literature on IPC to inform the structure of the pathway. Theoretical foundations were drawn from Reeves et al. (2010), Barr et al. (2006), and Tsakitzidis & Van Royen (2018) [25, 26, 64].

### Patient journey and service blueprint

HCPs created a timeline integrating patient journey contact points with the service blueprint of the desired care process. The different group timelines were then consolidated into a single 12-month and 3-month overview.

From the perspective of HCPs, every professional could and should play a role in detecting and screening for risk of malnutrition and sarcopenia if they have the necessary skills and knowledge. An exception is sarcopenia screening using handgrip strength assessment, as defined by the EWGSOP2 criteria, due to the unavailability of handgrip dynamometers for many professionals. Physiotherapists often use this device in practice, whereas other HCPs typically do not. However, from the professionals’ viewpoint, any HCP can perform the chair stand test [65], the Dutch SNAQ 65+ [66] and the Dutch PG-SGA Short Form for malnutrition screening [67].

Additionally, HCPs emphasised that the risk of malnutrition and sarcopenia can also be detected by older adults themselves, their social network, and social workers. This broader awareness and involvement could enhance early identification and intervention.

HCPs also acknowledged the need for improved coordination to streamline care delivery. They discussed the possibility of aligning schedules to determine who would visit the older adult during a specific week and explored opportunities to delegate certain tasks to other HCPs where appropriate, promoting better coordination and reducing redundancy. Examples of these interchangeable tasks include providing nutrition and physical activity advice, administering nutritional screening questionnaires to patients, motivating and supporting patients in improving their eating and drinking habits, and encouraging physical activity and exercise. Additionally, incorporating brief repetitions of exercise into routine consultations could be integrated into the roles of various HCPs, such as nurses, GPs, assistants and dietitians. It was emphasised that successfully executing these shared tasks requires effective communication and trust among HCPs.

### Prototyping

In the develop phase, two online co-design sessions were held, during which concept versions of the prototype were presented to HCPs. The workflow and its presentation were refined. Validated screening tools were specified, and actions for negative versus positive screening results were clarified. Roles were defined, and the detection cards, task-allocation formats, collaboration agreements, and arrangements for regular team evaluations were refined to improve clarity, structure, and accessibility.

Additionally, feedback on the detection card was obtained from older adults and from family members or informal caregivers in their networks. The detection card was generally clear, and feedback led to minor changes to wording and layout to improve readability.

## Final prototype: interprofessional care pathway for (risk of) malnutrition and sarcopenia

The interprofessional care pathway provides a structured workflow for detecting, screening, and managing malnutrition and sarcopenia in community-dwelling older adults [Additional file 4]. The process begins with identifying potential signs of malnutrition or sarcopenia. If no signs are observed, general advice on nutrition and physical activity is recommended, while the presence of signs prompts further screening by a professional. Validated tools, such as the SNAQ 65+ and PG-SGA Short Form for assessing the risk of malnutrition, as well as the chair stand test and handgrip strength test for detecting muscle strength loss, are recommended in the care pathway. A negative screening result is followed by general health recommendations and rescreening after three months. In contrast, a positive result indicates the need for treatment, involving dietary treatment, strength and functional training provided by a dietitian and physiotherapist, with outcomes evaluated three months later.

The care pathway emphasises IPC, ensuring that multiple disciplines work together through shared responsibility, knowledge exchange, a shared vision, and clearly defined roles. The approach to care is person-centred and integrated, with a designated point of contact for patients, a shared treatment plan, and continuous communication among professionals to make and adjust agreements on tasks and responsibilities within the collaboration.

Moreover, additional tools or formats are included to support the pathway, such as detection cards for the early identification of (risk of) malnutrition and sarcopenia, aimed at assisting HCPs, informal caregivers, older adults, and their social networks. The pathway also includes formats for documenting roles, task allocation, and agreements, ensuring IPC among disciplines. The pathway embeds a format for regular team evaluations to monitor progress and make necessary adjustments.

## Discussion

This study aimed to co-design a prototype interprofessional care pathway tailored to community-dwelling older adults with (risk of) malnutrition or sarcopenia. The prototype interprofessional care pathway provides a structured approach for detecting, screening, and managing (risk of) malnutrition and sarcopenia in community-dwelling older adults. It emphasises collaborative, person-centred care involving multiple disciplines working together with clear roles and goals.

Beyond producing the pathway, the co-design process generated insights beyond the interprofessional care pathway itself, particularly for the treatment of older adults with (risk of) malnutrition and sarcopenia. Interprofessional journey mapping revealed fragmented information flows, overlapping and distinct patient contact points, and inconsistent follow-up care, highlighting opportunities to reduce redundancies and streamline hand-offs. Teams used the artefacts to assign who does what, when, and with whom, converting implicit expectations into clear role agreements. Participants also reported greater awareness of each other’s roles, noting where escalation criteria and documentation standards were needed to support task substitution without compromising quality. Collectively, these lessons show that structured IPC supports practical pathway development and deepens understanding of interprofessional dynamics.

The interprofessional care pathway may help strengthen process coherence by clarifying professional roles and providing structured tools to support more coordinated workflows. For example, the task division template outlines suggested responsibilities across disciplines, helping teams allocate actions such as screening, referral, and follow-up. Meeting templates provide space to document decisions, role assignments, and interprofessional agreements. Shared screening instruments and care plans may enhance content coherence by supporting a mutual understanding of clinical thresholds and intervention strategies. Together, these tools surface context-specific cues for HCPs, which potentially can activate interprofessional identity and support timely, collaborative action [41, 42]. The pathway may also help social coherence by supporting regular team reflection and fostering shared expectations among professionals. Findings from other studies further underscore the importance of strong interprofessional relationships and coherent workflows in enhancing care quality [68].

These findings align with international efforts to standardise integrated care. The interprofessional care pathway reflects principles from the WHOs Integrated Care for Older People (ICOPE): Guidance for Person-Centred Assessment and Pathways in Primary Care [69], specifically its emphasis on timely detection of (risk of) malnutrition and loss of muscle strength, as well as on collaboration across disciplines. Our pathway supports early identification of malnutrition and sarcopenia. It combines recognition of early signs and risk factors with detection cards and validated screening tools to identify both conditions and those at risk. The interprofessional care pathway also mirrors ICOPEs emphasis on IPC and person-centred care through shared decision-making, role clarity, and team-based planning. While ICOPE provides a broad conceptual framework across multiple domains of functional decline, our pathway translates these principles into a locally applicable format tailored to (risk of) malnutrition and sarcopenia. As such, it may represent a meaningful step toward translating ICOPE principles into routine primary care practice.

We adopted the Double Diamond model in our co-design process because it offers a clear visualisation of human-centred design and aligns with the underlying principles of Design Thinking [44, 70, 71]. Design Thinking (Empathise, Define, Ideate, Prototype, Test) also organises iterative cycles of divergence and convergence, but places greater emphasis on early empathy and rapid prototyping. The Double Diamond offers a simplified representation of this logic. Co-design is also frequently conducted using Experience-Based Co-Design (EBCD), a form of participatory action research [72]. In contrast to EBCD, we did not undertake ethnographic data collection. Instead, we applied service design methods aligned with EBCD to make frontstage and backstage work visible. A novel aspect of our approach was the embedding of context-specific cues that could trigger action within one’s role (professional identity) and prompt acting together (interprofessional identity triggers). This design-research orientation fits complex problems that require stakeholders and disciplines to reshape how they collaborate. While EBCD is chiefly used for service redesign, its potential for intervention design in research and policy contexts has been noted; our study applies these principles to an interprofessional care pathway [72].

### Strengths and limitations

The iterative nature of the Double Diamond model supported the study’s rigour and relevance. This approach, in which data collection was carried out through multiple data points throughout the process, enabled continuous refinement of the care pathway based on participant feedback and insights gathered throughout the study. The active involvement of a diverse group of HCPs, patient personas, and an older adult’s participation ensured that the care pathway was grounded in real-world primary care experiences. By integrating perspectives from multiple disciplines and the end-user of care, the co-design process enhanced the pathway’s relevance and increased the likelihood of practical acceptance and implementation.

However, the study also has limitations. Firstly, a potential source of bias is the self-selection of participating HCPs, who may have had a more substantial interest in IPC than the broader primary care workforce. On the other hand, participating HCPs demonstrated high interprofessional identity and competence, which likely enriched the co-design process. Prior work suggests that HCPs with stronger interprofessional identity generate more practical solutions and collaborate more effectively across professional boundaries [32]. Secondly, only one older adult with (risk of) malnutrition and/or sarcopenia was directly involved in the co-design process, which limits the diversity of patient perspectives represented. This limited involvement reflects a broader issue in gerontological research, in which health-related burdens often limit participation and raise concerns about representativeness [73]. To help address this, personas developed from prior interviews and focus groups were used during the sessions to embed patient input and enhance the transferability of findings. While this study focused primarily on back-stage processes, it is equally important to examine how these arrangements translate into front-stage care experiences. Future work should therefore involve older adults more directly and consider including caregivers to complement patient perspectives on front-stage aspects of IPC.

### Implications for practice and future research

The interprofessional patient journey timeline visualisation during the co-design sessions proved to be a valuable process mapping tool [Additional file 1]. It offered HCPs a clear overview of when and how collaboration occurs, supporting reflection on roles, coordination, and inefficiencies. This approach deepened the understanding of complex care processes and informed improvements aligned with both patient and organisational needs [74]. Based on our co-design experience, we recommend further exploration of its practical application to support shared reflection, clarify professional roles, and identify coordination gaps.

Previous work shows that conversational topics can function as interprofessional identity triggers that increase speaking up in mixed-profession groups, and that contextual cues can act as identity triggers that encourage collaborative behaviours [42]. Building on this, future research should test the pathway’s embedded interprofessional identity triggers quantitatively to determine whether they function as intended and whether variation in interprofessional identity better explains adherence to evidence, greater professional effort, and improved collaboration. Linking these triggers to observable outcomes would provide a mechanism-based (how-and-why) explanation for why interprofessional care pathways could improve IPC.

Future research should also assess the feasibility, acceptability, appropriateness, and impact of the developed care pathway in line with the framework for developing and evaluating complex interventions [75]. Rather than offering a fixed blueprint, the care pathway should be viewed as flexible and requires local adaptation [75, 76]. Implementation is likely to benefit from a co-design approach, in which HCPs explore how to integrate the pathway into existing care processes and team routines.

These considerations also prompt reflection on the optimal design of care pathways for older adults. Rather than structuring pathways around single conditions such as (risk of) malnutrition or sarcopenia, an alternative approach may involve organising care to reflect the multifaceted and often interrelated health needs associated with ageing. The literature on person-centred care increasingly supports integrated, needs-based models over condition-specific protocols, particularly in populations with complex and overlapping health issues [69, 77]. However, rather than viewing needs-based and disease-specific approaches as mutually exclusive, future pathway development may benefit from modular designs that incorporate standardised interprofessional processes while retaining flexibility to address specific clinical contexts.

Successful implementation also requires strong leadership and organisational commitment, including the active involvement of decision-makers, alignment with institutional priorities, and the allocation of sufficient resources to sustain change [78]. Addressing barriers such as limited time, fragmented reimbursement, and lack of digital infrastructure is essential to ensure the pathway’s integration into daily primary care practice [49].

Although developed within the Dutch primary care context, the pathway’s structured approach may offer a transferable basis for other primary care systems, provided that it is co-designed with local stakeholders and end-users to ensure contextual fit. While the clinical content of the interprofessional care pathway is specific to (risk of) malnutrition and sarcopenia, the process components are generic and potentially transferable to other shared problem domains that require interprofessional care. Transfer will require condition-specific adaptation, for example, of screening criteria, professional roles, and intervention content.

## Conclusion

This study presents the co-design of an interprofessional care pathway for (risk of) malnutrition and sarcopenia in community-dwelling older adults. The pathway was developed through an iterative co-design process involving thirteen healthcare professionals, including district nurses, dietitians, physiotherapists, general practice assistants, dementia case managers, general practitioners and a geriatric specialist, and was informed by the perspectives of older adults. It integrates detection tools, screening instruments, and interprofessional treatment strategies. It also includes practical formats to support role clarification, task allocation, and ongoing team evaluation.

Future research should evaluate the pathway’s feasibility, acceptability, and appropriateness in routine practice, as well as its impact on patient outcomes.

## Electronic Supplementary Material

Below is the link to the electronic supplementary material.

Supplementary Material 1

Supplementary Material 2

Supplementary Material 3

Supplementary Material 4

## Data Availability

Anonymised data and metadata, including co-design session outputs, are available in the supplementary materials, with additional materials available upon reasonable request from the corresponding author.
