# Supplementary figures and images for: Co-designing an interprofessional care pathway for (risk of) malnutrition and sarcopenia in community-dwelling older adults
Source: BMC Health Serv Res. 2026 Jan 20;26:245. doi: 10.1186/s12913-026-14047-7 (PMC12903304; doi:10.1186/s12913-026-14047-7)

Interprofessional patient journey map and service blueprint

12-month timeline

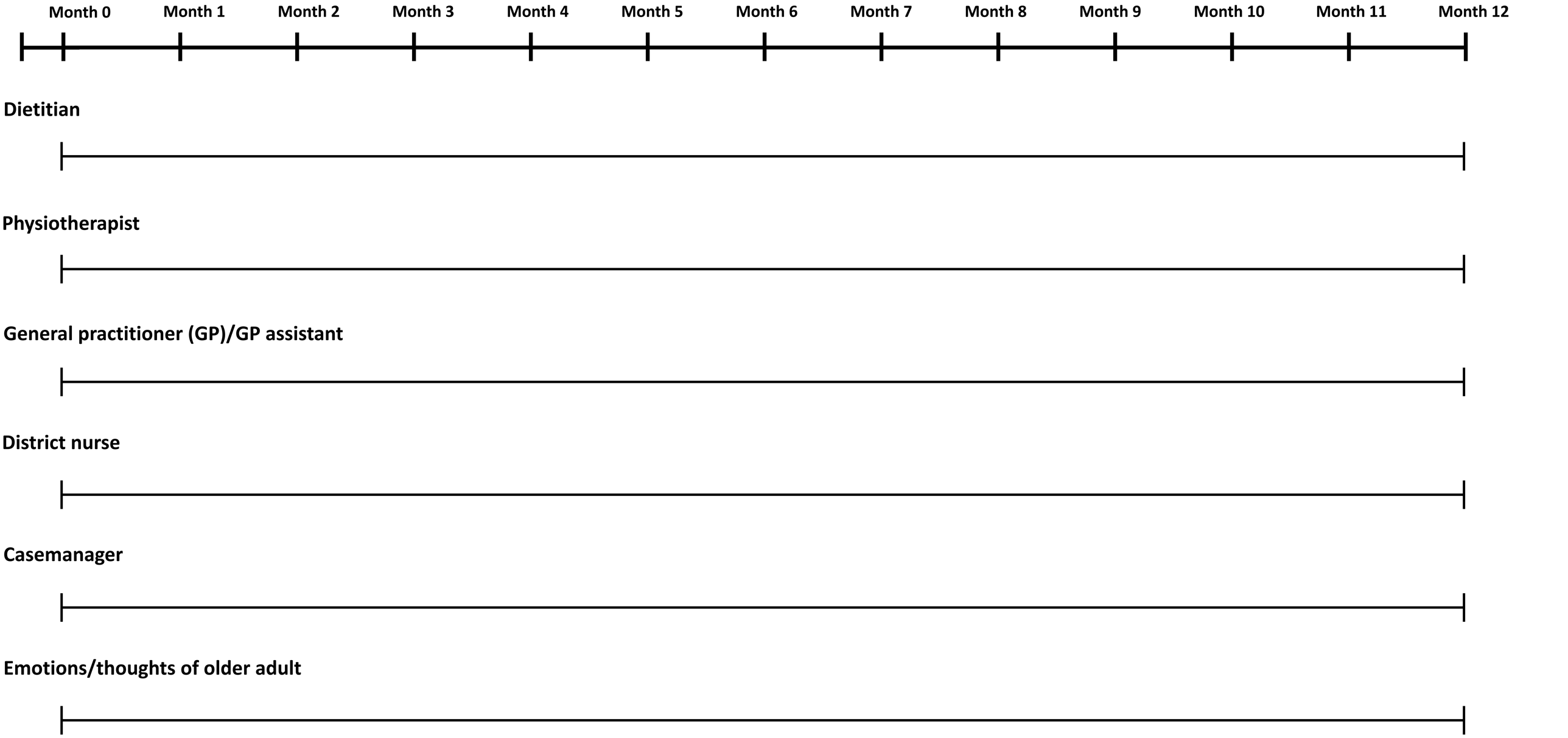

Supplement: Supplementary file 1 — Supplementary Material 1 [file 12913_2026_14047_MOESM1_ESM.pdf]
