# Supplementary material for: Co-designing an interprofessional care pathway for (risk of) malnutrition and sarcopenia in community-dwelling older adults
Source: BMC Health Serv Res. 2026 Jan 20;26:245. doi: 10.1186/s12913-026-14047-7 (PMC12903304; doi:10.1186/s12913-026-14047-7)
Supplement: Supplementary file 2 — Supplementary Material 2 [file 12913_2026_14047_MOESM2_ESM.pdf]

## Version 1 Persona A

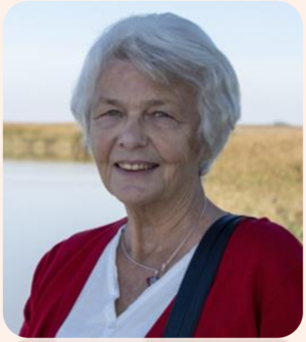

Image from: Pixabay

### Biography

Jannie grew up in a small village, where she completed her primary school education. Later, she moved to a nearby town for love. Choosing to stay at home, she devoted herself to raising her children and, later, caring for her grandchildren.
Her daily life revolves around household chores, gardening, and enjoying the simple things in life. Jannie has little experience with healthcare or social services, but she now requires some support due to declining mobility and health issues. Her husband passed away two years ago. She prefers to continue doing everything as she always has, such as working in the garden. However, this has led to several falls.

### Demographics

| | |
|---|---|
| Age | 83 |
| Gender | Female |
| Education/ profession | Primary school education |
| Family | Three adult children |
| Living situation | Lives alone |
| Diagnosis | Malnutrition and sarcopenia |

### Emotions/thoughts/concerns

Jannie regrets that healthcare has changed. She no longer knows her general practitioner well, and he is always busy. She often feels that her problems are not serious enough to warrant a call, so she postpones making one. However, she is satisfied with the care she receives.

### Wishes

Jannie does not like having unknown people visit her. She prefers familiar faces. She does not want much contact with healthcare providers, only when necessary. She prefers to visit the practice in person. She prefers personal contact and would rather be called by the practice nurse for results.

### Needs

She cannot log into her online dossier herself, but does not feel that this is necessary.

**Version 1 Persona B**

**Biography**
André began his career as an electrician. Together with his wife, he raised four children. Their children now live across the Netherlands. Over the past two years, André has experienced increasing physical complaints and has also become more forgetful. Despite these health problems, André is determined to maintain his independence. He lives with his partner, who is also his informal carer. They share a close and loving bond, but André's physical and mental health has been deteriorating. As a result, more and more of André's responsibilities fall on his wife's shoulders.

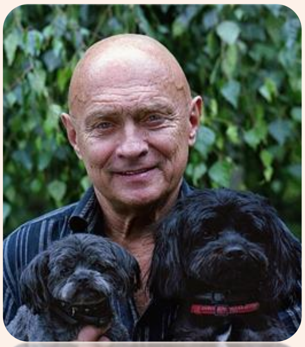

Image from: Pixabay

**Demographics**

| | |
|---|---|
| Age | 72 |
| Gender | Male |
| Education/ profession | Retired electrician |
| Family | Four adult children |
| Living situation | Lives with partner |
| Diagnosis | Sarcopenia |

**Emotions/thoughts/concerns**
André is worried about the future. He fears that the caregiving tasks will become too overwhelming for his wife in the long term.

**Wish**
André wishes to remain living at home with his wife for as long as possible. He would also like to go out occasionally, for example, for a walk in nature or a meal at a nice restaurant.

**Needs**
André and his wife need structure to help organise their daily lives. Additionally, they require more information and support to manage André's health problems.

**Version 1 Persona C**

**Biography**
Christa worked in the library for many years before retiring. Christa loves books and reading. She spends much time reading stories and browsing through books, discovering new authors. She enjoys walking in nature and participates in a reading club in her neighbourhood. During the time when she was ill, she lost much weight. Her weight has not returned to normal. Additionally, she lacks the energy to take part in social activities, such as the reading club. In the hospital, Christa had a dietitian at her bedside, but she was not happy with the care she received.

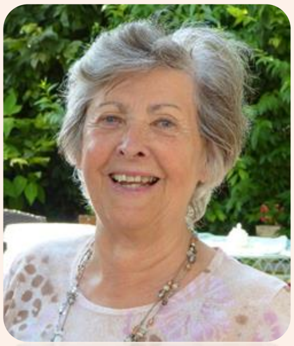

Image from: Pixabay

**Demographics**

| | |
|---|---|
| Age | 71 |
| Gender | Female |
| Education/ profession | Retired librarian |
| Family | No children |
| Living situation | Lives with partner |
| Diagnosis | Malnutrition |

**Emotions/thoughts/concerns**
Christa sometimes feels she is not treated equally in conversations with healthcare professionals. These experiences cause her to worry about whether she is receiving the right care to regain her former self

**Whishes**
Christa wishes to be able to enjoy eating a small meal again and regain her previous energy levels. She values her independence and would like to be able to determine how her life unfolds.

**Needs**
Christa wants to be included in decisions regarding her health and would like to receive information about her diagnosis.

**Version 1 Persona D**

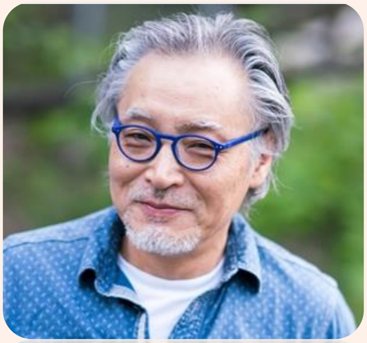

Image from: Pixabay

**Biography**

William was a self-employed entrepreneur before retiring. He remains involved in his former business, which his son now manages, and occasionally works there. He enjoys reading, golfing, and travelling to exotic destinations.

Since last year, William's life has drastically changed due to illness. He finds it more difficult to move and often feels too tired to engage in activities he once enjoyed. He now has numerous appointments with healthcare providers, both within and outside the hospital. Since his illness began, William has not visited his former business.

**Demographics**

| | |
|---|---|
| Age | 67 |
| Gender | Male |
| Education/ profession | Higher education, retired entrepreneur |
| Family | One son |
| Living situation | Lives alone |
| Diagnosis | Malnutrition and sarcopenia |

**Emotions/thoughts/concerns**

William is frustrated that he often has to repeat the same information to different healthcare providers. Furthermore, he feels sad that he has lost his connection with the business world. He is also slowly losing control over his life, which affects his emotional state.

**Wishes**

William wishes for more rest in his schedule. The numerous healthcare appointments are sometimes overwhelming for him.

**Needs**

William needs clear appointments with healthcare providers and clear communication and coordination between providers.
