# Supplementary material for: Co-designing an interprofessional care pathway for (risk of) malnutrition and sarcopenia in community-dwelling older adults
Source: BMC Health Serv Res. 2026 Jan 20;26:245. doi: 10.1186/s12913-026-14047-7 (PMC12903304; doi:10.1186/s12913-026-14047-7)
Supplement: Supplementary file 3 — Supplementary Material 3 [file 12913_2026_14047_MOESM3_ESM.pdf]

Version 2 Persona A

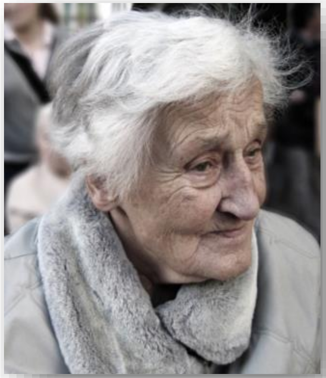

Image from: Pixabay

"I no longer feel like I know my general practitioner. There's no time for a friendly chat like there used to be, and I miss that."

Biography

Jannie grew up in a small village, where she completed her primary education. She spent her life caring for her children, and her days have always revolved around housekeeping, gardening, and enjoying the simple things in life. Jannie has limited experience with professional care or support but now requires assistance. Her husband passed away five years ago from cardiac arrest. Jannie prefers to keep doing everything she used to, such as gardening, but this has caused her to fall several times.

Demographics

Age	91
Gender	Female
Education/ profession	Primary school education
Family	Three adult children
Living situation	Lives alone
Diagnosis	Malnutrition and sarcopenia

Emotions/thoughts/concerns

Jannie regrets that healthcare has changed. She no longer knows her general practitioner well, and he is always busy. She often feels that her problems are not serious enough to warrant a visit to the general practitioner, so she postpones seeking help. However, she is satisfied with the care she receives.

Needs and preferences

Jannie prefers personal contact with healthcare professionals. Since she lives in a remote area, she prefers appointments at home. Additionally, Jannie struggles to understand information provided by healthcare professionals. She would appreciate more explanation and time to help her understand this information better.

Version 2 Persona B

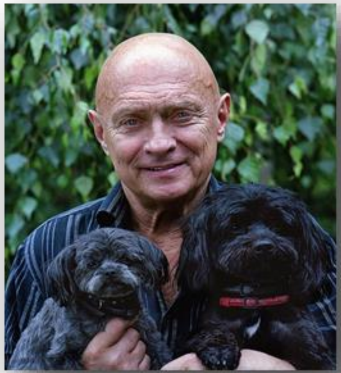

Image from: Pixabay

"We want to stay at home together as long as possible, but caring for me is getting harder for my wife."

Demographics

Age	72
Gender	Male
Education/ profession	Retired electrician
Family	Four adult children
Living situation	Lives with partner
Diagnosis	Sarcopenia

Biography

André began his career as an electrician. Together with his wife, he raised four children. Their children now live across the Netherlands. Over the past two years, André has experienced increasing physical complaints and has also become more forgetful. Despite these health problems, André is determined to maintain his independence. He lives with his partner, who is also his informal carer. They share a close and loving bond, but André's physical and mental health has been deteriorating. As a result, more and more of André’s responsibilities fall on his wife’s shoulders.

Emotions/thoughts/concerns

André is worried about the future. He fears that the caregiving tasks will become too overwhelming for his wife in the long term.

Needs and preferences

André wishes to remain living at home with his wife for as long as possible. He would also like to go out occasionally, for example, for a walk in nature or a meal at a nice restaurant. André and his wife need structure to help organise their daily lives. Additionally, they require more information and support to manage André’s health problems.

Version 2 Persona C

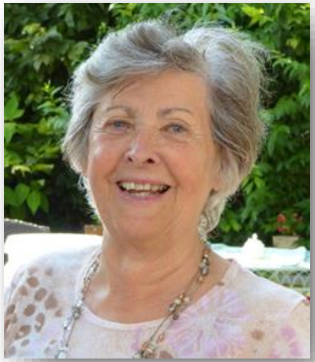

Image from: Pixabay

"I want to be involved in discussions about what is best for me. Healthcare professionals should talk with me, not about me."

Biography

Christa worked for many years in a library before retiring. She loves books and reading, spending much of her time reading stories, magazines, and discovering new authors. She enjoys walks in nature and participates in a local book club. During the period when she was unwell, Christa lost a significant amount of weight, and her appetite has not fully returned. Additionally, she does not yet have enough energy to resume social activities such as her book club.

Demographics

Age	80
Gender	Female
Education/ profession	Retired librarian
Family	No children
Living situation	Lives with partner
Diagnosis	Malnutrition

Emotions/thoughts/concerns

Christa sometimes feels she is not treated equally in conversations with healthcare professionals. These experiences cause her to worry about whether she is receiving the right care to regain her former self. Her concerns are further complicated because her partner does not always eat regular meals, occasionally leading to tensions in their household.

Needs and preferences

Christa wishes to enjoy a simple meal again and regain her previous energy levels. She hopes to be actively involved in decisions about her health and wants more information about her diagnosis.

Version 2 Persona D

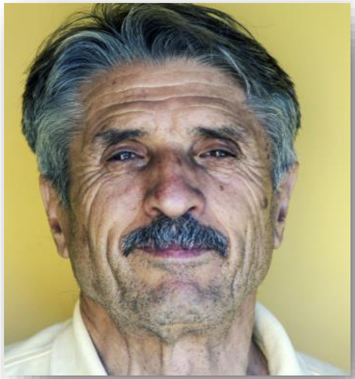

Image from: Pixabay

"I have to repeat my story to different healthcare professionals often, which frustrates me."

Biography

Mehmet was a self-employed entrepreneur. He remains somewhat involved in his former business, now run by his son, and contributes occasionally. However, his life has changed drastically due to illness since last year. He experiences difficulty with mobility and often feels too tired to engage in activities. Mehmet has numerous appointments with healthcare providers within and outside the hospital. Since the onset of his illness, he has not returned to his former business.

Demographics

Age	67
Gender	Male
Education/ profession	Higher education, retired entrepreneur
Family	One son
Living situation	Lives alone
Diagnosis	Malnutrition and sarcopenia

Emotions/thoughts/concerns

Mehmet feels frustrated because his healthcare providers often lack awareness of each other's activities. As a result, he feels compelled to repeatedly share the same information. He finds this challenging as Dutch is not his first language, and his proficiency in English is limited. The feeling of not always being understood weighs heavily on him. In addition, Mehmet feels a sense of loss over his connection to his former business.

Needs and preferences

Mehmet desires more structure and calm in his schedule, as the frequent healthcare appointments can feel overwhelming. He needs clear communication with healthcare providers and well-coordinated agreements between them.
