# Supplementary material for: Co-designing an interprofessional care pathway for (risk of) malnutrition and sarcopenia in community-dwelling older adults
Source: BMC Health Serv Res. 2026 Jan 20;26:245. doi: 10.1186/s12913-026-14047-7 (PMC12903304; doi:10.1186/s12913-026-14047-7)
Supplement: Supplementary file 4 — Supplementary Material 4 [file 12913_2026_14047_MOESM4_ESM.pdf]

Interprofessional care pathway for (risk of) malnutrition and sarcopenia in community-dwelling older adults

This version is translated from Dutch

Version 1.0 – July 2025

SANDRA D. BOXUM, JAN-JAAP REINDERS, MANON G. A. VAN DEN BERG, MICHAEL TIELAND, PHILIP J. VAN DER WEES, HANS DRENTH, HARRIËT JAGER-WITTENAAR.

Preconditions for interprofessional collaboration in the treatment of (risk of) malnutrition and sarcopenia in community-dwelling older adults

Team development

- Multiple disciplines involved
- Shared responsibility
- Knowledge of each other's expertise
- Developing a shared vision
- Equality among disciplines
- Agreement on roles and task allocation
- Team evaluation

Interprofessional care delivery

- Integrated approach
- Client-centered approach
- One point of contact for the client
- Joint treatment plan
- Regular communication, coordination, and adjustments (client-centred)

Clinical suspicion
Are there any signs that could indicate (risk of) malnutrition and/or sarcopenia?

- Detection guide for professionals
- Detection guide for older adults, informal carers, and the social network to help them recognise potential signs.

No signs →

Offer general guidance on nutrition and physical activity.

Stay alert for signs.

↓ Yes, signs were detected

Screening
Use validated screening tools for (risk of) malnutrition (PG-SGA SF or SNAQ 65+) and sarcopenia (handgrip strength and chair stand test).

→ Negative ←

Offer general guidance on nutrition and physical activity.

Screen again in three months for (risk of) malnutrition and sarcopenia.

↓ Positive

Treatment for (risk of) malnutrition and sarcopenia

Interprofessional collaboration

Dietary treatment

Strength and functional training

Evaluation after three months

Tables of contents

1. Background ... 6
  1.1 Rationale ... 6
  1.2 Objective ... 6
  1.3 Definitions ... 6
  1.4 Users of the care pathway ... 7
  1.5 Target population ... 7
  1.6 Use of the care pathway ... 7
  1.7 Development of the care pathway ... 7
2. Preconditions for interprofessional management of (risk of) malnutrition and sarcopenia ... 9
  2.1 Team composition ... 9
  2.2 Shared vision ... 9
    2.2.1 What is a shared vision? ... 9
    2.2.2 How do you develop a shared vision? ... 9
  2.3 Equality among disciplines ... 10
  2.4 Knowledge of each other's expertise ... 10
  2.5 Shared responsibility ... 10
  2.6 Role and task allocation ... 10
    2.6.1 Roles in the team ... 10
    2.6.2 Agreements on task distribution ... 10
  2.7 Regular meetings ... 11
    2.7.1 Regularly informing, coordinating, and adjusting (client-focused) ... 11
    2.7.2 Team evaluation ... 11
  2.8 Integrated approach to malnutrition and sarcopenia ... 11
  2.9 Client-centred at a collective level ... 11
  2.10 One point of contact for the client ... 13
  2.11 Establishing a single joint treatment plan ... 13
3. Clinical suspicion, screening, and diagnostics ... 14
  3.1 Clinical suspicion ... 14
  3.2 Screening ... 14
    3.2.1 Negative screening result ... 14
    3.2.2 Positive screening result ... 14
    3.2.3 General advice regarding nutrition and exercise ... 14
  3.3 Diagnostics ... 16
4. Dietary treatment, physical and functional training ... 17

4.1 Treatment guidelines ... 17
4.1.1 Allied healthcare guideline for frail older adults ... 17
4.1.2 Guidelines for dietary treatment ... 17
4.1.3 Guideline for physical and functional training ... 17
4.2 Evaluation of treatment ... 18
4.2.1 Recommended assessments and measurements for malnutrition ... 18
4.2.2 Recommended assessments for sarcopenia ... 18
4.3 Monitoring and follow-up ... 19
5. References ... 20
Appendix 1 Team evaluation with the QuickScan interprofessional team collaboration (translated from Dutch) ... 23
Appendix 2 Detection guide for older adults, caregivers, and the social network ... 27
Appendix 3 Detection guide for professionals ... 29
Appendix 4 Screening and diagnosing malnutrition ... 31
PG-SGA Short Form ... 31
SNAQ 65+ ... 31
GLIM-criteria ... 32
Appendix 5 Screening and diagnosing sarcopenia ... 33
EWGSOP2-criteria ... 33
Chair stand test ... 33
Handgrip strength ... 33
Bioelectrical impedance analysis (BIA) ... 33
Appendix 5 Guidelines for team agreements ... 35
Appendix 6 Guidelines for documenting team agreements per client ... 36
1. Client care coordinator ... 36
2. Involved professionals ... 36
3. Treatment goals ... 36
4. Interventions and coordination ... 36
5. Task coordination and redistribution ... 36
Appendix 7 Guidelines for client meetings ... 37
Step-by-step plan for client discussions ... 38
Form Client discussion ... 38
Recorded agreements during client discussions ... 39

1. Background

1.1 Rationale

Malnutrition and sarcopenia are characterised by a severe loss of muscle mass and muscle strength. Both conditions can lead to falls, more frequent visits to the general practitioner, and an increased chance of admission to hospitals, care homes, or nursing homes [1]. Among community-dwelling older adults, malnutrition and sarcopenia are common, occurring in 20-40% [2] and 10-40% [3], respectively. With the increasing ageing population and comorbidity, the risk of malnutrition and/or sarcopenia among community-dwelling older adults will further increase in the coming years [4].

Preventing (risk of) malnutrition and sarcopenia in community-dwelling older adults is of great importance, given the serious consequences for health, independent functioning, well-being, and quality of life. Research shows that a combined diet and exercise intervention is most effective for improving muscle strength, muscle mass, and physical functioning in older adults [5, 6].

Due to the complexity of malnutrition and sarcopenia, it is necessary to combine various areas of expertise to address these conditions effectively. Interprofessional collaboration can play an essential role in this. Interprofessional collaboration can improve the quality of care for clients and the job satisfaction of healthcare providers [7, 8]. Moreover, interprofessional collaboration leads to higher overall client satisfaction and can even reduce healthcare utilisation [9].

Through interprofessional collaboration, various disciplines, such as general practitioners, dietitians, physiotherapists, and community nurses, can combine their knowledge and work together on a treatment plan tailored to the needs of the older adult client with (risk of) malnutrition and sarcopenia.

1.2 Objective

This care pathway aims to optimise the approach to (risk of) malnutrition and sarcopenia in community-dwelling older adults through interprofessional collaboration. By optimisation, we strive for effective, efficient, and enjoyable care for all involved.

1.3 Definitions

Malnutrition is described as an acute or chronic condition in which a deficiency or imbalance of energy, protein, and other nutrients leads to measurable adverse effects on body composition, functioning, and clinical outcomes [10].
Sarcopenia is described as a skeletal muscle disorder characterised by progressive and systemic loss of skeletal muscle mass and strength, with a risk of adverse outcomes such as physical limitations, reduced quality of life, and premature death [11].
Interprofessional collaboration refers to the collaborative effort among health and/or social care providers from different professional backgrounds. These individuals work together toward shared goals, contributing their unique expertise and skills. This approach involves the joint planning, implementation, and evaluation of care, with shared knowledge and responsibilities. [12-14].
Interprofessional collaboration differs from other common forms of collaboration, such as the often-mentioned multidisciplinary collaboration. Interprofessional collaboration emphasises the integration of different fields. In other forms of collaboration, tasks are

usually divided among different professionals, and there is often little coordination regarding the content of the work. In contrast, interprofessional collaboration involves professionals actively integrating their expertise and skills to jointly develop a treatment plan, preferably with the client and their social network. This involves close coordination and focuses on jointly addressing the client's care needs.

Table 1 Comparison between multidisciplinary and interprofessional collaboration [15]

Aspect	Multidisciplinary	Interprofessional
Multiple disciplines are involved in care	Yes	Yes
Consultations and meetings	Occasionally	Yes
Shared vision	Possible	Yes
Equality of disciplines	Possible	Yes
Joint care plan	No	Yes
Agreements on task distribution	Possible	Yes
Knowledge of each other's field	Possible	Yes
Holistic approach	No	Yes
Client-centred	Yes, at a monodisciplinary level	Yes, at joint level
One point of contact for the citizen/client/patient	No	Yes
Joint responsibility for the implementation of the care plan	No	Yes

1.4 Users of the care pathway
This care pathway is intended for all primary care professionals involved in recognising and managing (risk of) malnutrition and/or sarcopenia in older adults.

1.5 Target population
The care pathway applies to adults aged 65 and older with (risk of) malnutrition and/or sarcopenia.

1.6 Use of the care pathway
The care pathway primarily focuses on professionals who are generally involved in addressing (the risk of) malnutrition and/or sarcopenia in community-dwelling older adults. This includes the general practitioner, general practitioner assistant and/or medical assistant, dietitian, and (geriatric) physiotherapist. This group of diverse professionals can be expanded with professionals from other professions, such as a case manager (dementia/frail older adults), community nurse, social worker, occupational therapist, psychologist, or geriatrician. However, agreements on communication, information exchange, documentation, role distribution, responsibilities, and decision-making within the team must be systematically recorded. These agreements within the team can be documented using the guidelines in the appendices to tailor the care pathway to the local context of the team.

1.7 Development of the care pathway
The care pathway was developed in co-creation in the following four steps:

1. Preliminary exploration phase: The wishes and needs regarding the interprofessional approach to (risk of) malnutrition and sarcopenia in community-dwelling older adults,

from the perspectives of both older adults and healthcare professionals, were explored through group discussions and individual interviews. Subsequently, the current organisation of care for (risk of) malnutrition and sarcopenia was mapped out during meetings with professionals and with the help of a patient journey with an older person with malnutrition and sarcopenia.

2. Design phase: In meetings with professionals, the desired situation for the care pathway, as outlined by professionals, was discussed. Various perspectives from different disciplines were discussed during the sessions, which contributed to insights on how these disciplines can strengthen and complement each other in addressing (the risk of) malnutrition and sarcopenia.
3. Integration phase: The outcomes of steps 1 and 2 were then integrated with existing evidence-based guidelines for malnutrition and sarcopenia, resulting in a prototype care pathway.
4. Finalisation: The prototype care pathway was discussed in conversations or online meetings with professionals, community-dwelling older adults, and experts in malnutrition and sarcopenia. The input from these diverse groups led to further refinement and finalisation of the care pathway, considering the different perspectives and needs.

2. Preconditions for interprofessional management of (risk of) malnutrition and sarcopenia

Interprofessional collaboration involves engaging different disciplines through regular consultations, agreements on role and task distribution, sharing a common vision, recognising equality between disciplines, understanding each other's fields, and joint responsibility. At the client level, this means an integrated, client-centred approach at a joint level. This is achieved by appointing a single point of contact for the client and creating a joint treatment plan.

2.1 Team composition

The team addressing malnutrition and/or sarcopenia in community-dwelling older adults consists of professionals from various disciplines. The team typically includes a general practitioner, practice nurse/assistant, dietitian, and (geriatric) physiotherapist/exercise physiologist.

Depending on the cause and specific needs of the client, other professionals may also be involved:
Occupational therapist: Engage an occupational therapist for issues with daily activities and receive advice on home modifications and the use of aids.
Speech therapist: Consult a speech therapist for issues related to chewing and swallowing.
Community nurse and/or nursing assistant: Involve the community nurse and/or nursing assistant for the nursing care needs of the older adult.
Nurse specialist: Engage the nurse specialist for patients with complex care needs requiring specialised nursing interventions and expertise.
Case manager for frail older adults or dementia: Consult the case manager for complex care needs related to ageing or dementia.
Geriatrician: Consult the geriatrician for older adults with complex medical care needs when the regular treatment team cannot adequately provide the necessary care and expertise.
Dentist, dental hygienist: Contact a dentist or dental hygienist for eating problems caused by ill-fitting dentures or prostheses, poor oral hygiene, pain, irritation, or mouth damage.
Social domain professionals: Involve advisors for older adults, ambulatory counsellors, and domestic help for social and practical support for the older person and their caregivers.

2.2 Shared vision

2.2.1 What is a shared vision?

A shared vision is a shared set of goals, values, beliefs, and expectations that form the foundation for collaboration and informed decision-making. This shared vision helps create a sense of unity and purpose, ensuring everyone in the team is aligned and moving in the same direction.

2.2.2 How do you develop a shared vision?

To develop a shared vision, the core values and goals for addressing (risk of) malnutrition and sarcopenia should be collaboratively determined. Ideas and expectations should be shared and integrated into a cohesive vision. Document this vision and communicate it both

internally and externally. Embed the vision into daily practice and regularly evaluate progress. Be open to adjusting based on these evaluations.

Example core values and vision

Core values: Respect, collaboration, and commitment to the well-being of our clients.

Vision: Our team is dedicated to the health and quality of life of individuals aged 65 and older who are at risk of or experiencing malnutrition and sarcopenia. Through close collaboration between us as healthcare providers and our clients, we strive for optimal treatment that enhances both nutritional intake and muscle strength.

2.3 Equality among disciplines

Recognising equality between disciplines means valuing and respecting all involved disciplines for their unique contributions to the care process. This includes understanding that each discipline has valuable knowledge and expertise essential for achieving common goals. It is important to create a culture where all team members feel heard and valued, regardless of their background or discipline. This fosters open and respectful collaboration, where everyone contributes equally to the success of the shared vision.

2.4 Knowledge of each other's expertise

Knowing each other's expertise means that team members understand the roles, responsibilities, and expertise of other disciplines within the team. Understanding each other's fields allows team members to collaborate, communicate, and make joint decisions more effectively. Sharing and valuing knowledge about each other's fields contributes to a culture of respect, trust, and mutual understanding within the team. Exchanging experiences during joint case discussions, training, and actively exploring each other's guidelines can help to increase knowledge about each other's disciplines.

2.5 Shared responsibility

Shared responsibility means that each team member actively commits to the common goal. This requires a culture where professionals communicate openly, trust each other, and strive for continuous improvement.

2.6 Role and task allocation

2.6.1 Roles in the team

Each professional in the team contributes to the interprofessional treatment of the client. The dietitian is primarily responsible for the client's dietary treatment, while the physiotherapist is predominantly responsible for muscle and functional training. Each team member contributes to the combined treatment and shares responsibility for the treatment outcomes. The precise distribution of roles and responsibilities is determined in consultation at the start of the treatment, considering the client's specific needs and the team members' expertise.

2.6.2 Agreements on task distribution

Analysing tasks based on competence and availability allows the team to determine which professional is suited to a particular task. When one team member becomes overloaded or experiences busy periods, it is essential to redistribute tasks within the team. Transferring

tasks to other team members who are more available at that time can alleviate the burden, ensuring that all tasks related to malnutrition and sarcopenia are completed on time. Coordination on the content of the task is essential.

Examples of potentially interchangeable tasks include:

A. Providing nutritional advice to the client
B. Administering a malnutrition questionnaire to the client
C. Weighing the client
D. Motivating and supporting the client in improving their eating and drinking habits
E. Advising the client on physical activity
F. Motivating and supporting the client in improving their exercise routine
G. Making recommendations for suitable physical activities to promote muscle mass and strength
H. Performing exercises with the client to promote muscle mass and strength
I. Monitoring whether the client is sufficiently active or performing exercises

2.7 Regular meetings

Regular meetings include client discussions, where clients' needs and progress are discussed, as well as the evaluation of team member collaboration.

2.7.1 Regularly informing, coordinating, and adjusting (client-focused)

Client meetings aim to establish the individual client's treatment goals and regularly evaluate them. Additionally, work agreements are made around the individual client within the team, such as appointing a care coordinator [format work agreements per client]. During these meetings, agreements are documented, emphasising preparation, documentation, and feedback to the client.

2.7.2 Team evaluation

Team evaluation provides a valuable opportunity to identify bottlenecks and find solutions that improve collaboration. These meetings also foster knowledge exchange, enabling team members to share their expertise, discuss recent developments, and exchange best practices, thereby supporting continuous learning and improvement. The Dutch Quick Scan IPT, described in Appendix 1 [16] can be a helpful tool for evaluating team collaboration. Team evaluations can be administered at the outset to assess team functioning and are recommended for regular use, ideally every two to three months, to promote ongoing improvement and address any issues promptly.

2.8 Integrated approach to malnutrition and sarcopenia

An integrated approach means that all relevant health and well-being aspects are integrated into treating (risk of) malnutrition and sarcopenia. This includes dietary interventions, strength and functional training, medication, social support, and identifying and addressing underlying health problems.

2.9 Client-centred at a collective level

The client, possibly accompanied by family and/or social network, should be at the centre of the interprofessional collaboration. Using the participation ladder as a guide can help determine and promote the level of client involvement as a partner in the care process. This fits well with person-centred care, where the client is considered as a person receiving care

and an equal partner in the care process. The client must indicate the extent to which they want to be involved. This should occur at the beginning of the care process and be regularly updated throughout, as the client's needs and preferences may change over time. Using the participation ladder as a guide can help determine the level of client involvement.

The steps of the participation ladder [17, 18]

1. **Clients are informed:** At this first level of the participation ladder, clients receive information about their health condition, available treatment options, and other relevant aspects of their care.
2. **Clients are consulted**: At the second level, clients are asked for their opinions and preferences. Here, clients are consulted but do not yet actively participate in the decision-making process.
3. **Clients are advised**: At this level, clients are actively involved in designing and planning care. They can give their advice and share their opinions during, for example, care plan discussions. However, final decisions are made by their healthcare providers.
4. **Collaborating with the client**: At the fourth level, the relationship between the client and caregivers is characterised by equal collaboration, where both parties actively contribute to the care process. For example, clients can participate in a care team where they collaborate with their healthcare providers to make decisions about treatment plans, therapies, and other aspects of their care.
5. **Clients take control:** At the highest level of the participation ladder, clients are actively encouraged to take control of their care and well-being. This can be achieved by supporting the client in developing self-management skills and making their own decisions about their care, with the necessary support and guidance from caregivers.

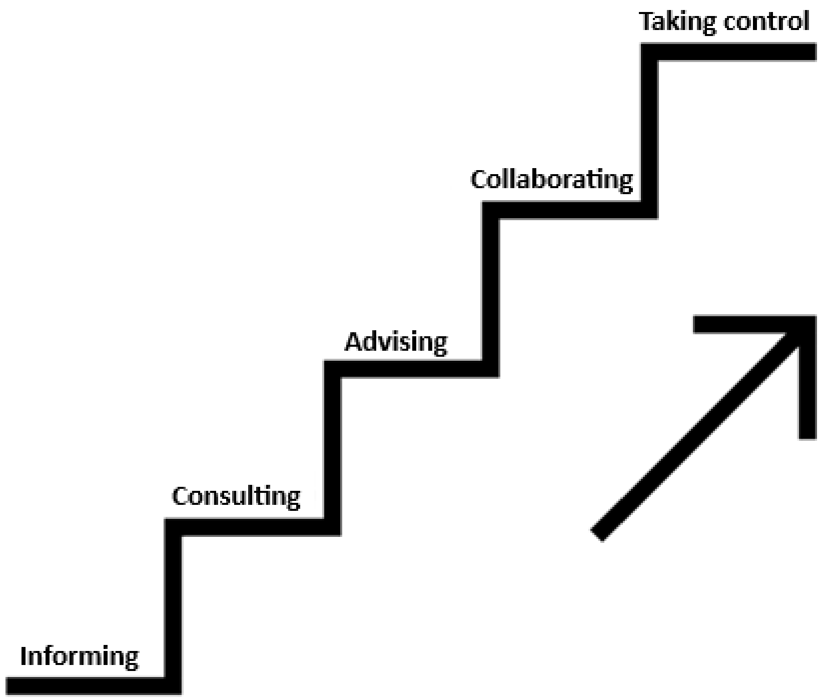

Figure 1 The participation ladder

2.10 One point of contact for the client

At the start of treatment, one professional is appointed as the care coordinator. Various professionals, including general practitioners, practice nurses or assistants, case managers, or community nurses, can fulfil this role. The care coordinator is responsible for coordinating care, monitoring the treatment plan, and serving as the point of contact for the client.

2.11 Establishing a single joint treatment plan

A treatment plan is jointly established during the first client meeting for each client with malnutrition and/or sarcopenia. This plan is regularly discussed and adjusted throughout the treatment to align with the client's changing needs and circumstances.

3. Clinical suspicion, screening, and diagnostics

3.1 Clinical suspicion

Detection involves recognising possible signs or symptoms of (risk of) malnutrition and/or sarcopenia. This detection can be performed by clients, caregivers, social networks, and all health and welfare professionals who come into contact with community-dwelling older adults. Using a detection guide can help identify possible signs or symptoms of (risk of) malnutrition and sarcopenia. A detection guide for older adults, caregivers, and the social network can be found in Appendix 2. The detection guide is available in Appendix 3 for health and welfare professionals.

We recommend providing general advice on nutrition and exercise to promote muscle strength and independent functioning, thereby preventing malnutrition and/or sarcopenia, both in the presence and absence of signs (see 3.2.3).

3.2 Screening

Screening is a standardised process to determine quickly if further diagnostics are needed. The Patient-Generated Subjective Global Assessment Short Form (PG-SGA SF) [19, 20] and the Short Nutritional Assessment Questionnaire 65+ (SNAQ 65+) [21] are recommended as screening tools for malnutrition in community-dwelling older adults. The implementation and reference values are described in Appendix 4. The handgrip strength measurement and chair stand test (also known as the Five Times Sit-to-Stand Test or the sit-to-stand test) are recommended to identify low muscle strength to screen for sarcopenia [11]. The handgrip strength measurement and chair stand test (also known as the Five Times Sit-to-Stand Test or the sit-to-stand test) are recommended as a screening tool for identifying low muscle strength in sarcopenia. The implementation and reference values for the handgrip strength and chair stand test are in Appendix 5. We do not recommend the SARC-F questionnaire for sarcopenia screening. Despite the good reliability of the SARC-F, its low to moderate sensitivity and moderate to high specificity make it less suitable for sarcopenia screening [22, 23].

3.2.1 Negative screening result

Provide general advice on good nutrition and exercise (see Section 3.2.3) for individuals with a negative screening result. Repeat the screening after three months to re-evaluate for (risk of) malnutrition and sarcopenia.

3.2.2 Positive screening result

With a positive screening result, treatment is initiated, and further diagnostic steps can be taken to confirm the diagnosis and develop an appropriate treatment plan. Refer to the team specialising in managing malnutrition and sarcopenia, or start the joint treatment if you are part of this team.

3.2.3 General advice regarding nutrition and exercise

The Wheel of Five (Dutch: De Schijf van Vijf) outlines dietary recommendations that are consistent with international standards set by the World Health Organisation (WHO) [24]. For adults aged 50 and older, the Wheel of Five advises choosing mainly foods from these groups, which can help limit the intake of foods with potentially adverse health effects. As people age, muscle mass decreases, reducing the body's energy needs from food. The need

for specific nutrients also changes with age. The general recommendations for adults aged 50 and older are shown in Table 2. These are general dietary recommendations; the advice may differ from the standard in cases of illness or disease.

Table 2 General recommendations according to The Wheel of Five for adults aged 50 and over [24]

Food group	Men 51–69 yrs	Women 51–69 yrs	Men >70 yrs	Women >70 yrs
**Vegetables**	250 g/day	250 g/day	250 g/day	250 g/day
**Fruit**	200 g/day	200 g/day	200 g/day	200 g/day
**Bread**	6–7 slices/day (210–245 g)	3–4 slices/day (105–140 g)	4–6 slices/day (140–210 g)	3–4 slices/day (105–140 g)
**Grains and potatoes**	4 servings/day (240 g)	3–4 servings/day (180–240 g)	4 servings/day (240 g)	3 servings/day (180 g)
**Fish**	Once/week (100 g)	Once/week (100 g)	Once/week (100 g)	Once/week (100 g)
**Legumes**	2–3 tablespoons/week (135 g)	2–3 tablespoons/week (135 g)	2–3 tablespoons/week (135 g)	2–3 tablespoons/week (135 g)
**Meat**	Max. 500 g/week (100 g/meal)	Max. 500 g/week (100 g/meal)	Max. 500 g/week (100 g/meal)	Max. 500 g/week (100 g/meal)
**Eggs**	2–3 eggs/week (100–150 g)	2–3 eggs/week (100–150 g)	2–3 eggs/week (100–150 g)	2–3 eggs/week (100–150 g)
**Nuts**	25 g/day	15 g/day	15 g/day	15 g/day
**Milk and dairy products**	3 servings/day (450 g)	3–4 servings/day (450–600 g)	4 servings/day (600 g)	4 servings/day (600 g)
**Cheese**	40 g/day	40 g/day	40 g/day	40 g/day
**Spreading and cooking fats**	65 g/day	40 g/day	55 g/day	35 g/day

**For grain products and potatoes, the advice is to eat at least half whole-grain products weekly.*
**For meat, the advice is to eat meat no more than five times a week, of which a maximum of three times red meat.*

The following exercise guidelines are recommended [25, 26]:

- Exercise is good, more exercise is better
- Exercise at least 150 minutes per week at moderate intensity, such as walking and cycling, spread over several days. Longer, more frequent, and/or more intense exercise provides additional health benefits
- Do muscle and bone-strengthening activities at least twice a week, combined with balance exercises for older adults
- Avoid prolonged sitting

Consider rescreening the client for (risk of) malnutrition and sarcopenia after three months.

3.3 Diagnostics

The diagnosis of malnutrition and/or sarcopenia is the joint responsibility of the dietitian and physiotherapist. The dietitian and physiotherapist coordinate their tasks regarding diagnosing malnutrition and/or sarcopenia. The Global Leadership Initiative on Malnutrition (GLIM) criteria [25] and the European Working Group on Sarcopenia in Older People (EWGSOP2) criteria [11] can be used to diagnose malnutrition and sarcopenia [11]. The GLIM criteria are described in Appendix 4, and the EWGSOP2 criteria are described in Appendix 5.

4. Dietary treatment, physical and functional training

4.1 Treatment guidelines

4.1.1 Allied healthcare guideline for frail older adults

The allied healthcare guideline for frail older adults (Dutch: De paramedische richtlijn voor kwetsbare ouderen) provides practical recommendations and guidelines regarding, among other things, malnutrition and sarcopenia [27]. The guideline is aimed explicitly at dietitians, physiotherapists, exercise physiologists, occupational therapists, skin therapists, and speech therapists. It is also relevant for caregivers and other healthcare professionals involved in the care of frail older adults. The guideline is applicable across all levels of care, including primary, secondary, and tertiary care. It emphasises the importance of collaboration between different allied healthcare professionals to optimise care for frail older adults.

4.1.2 Guidelines for dietary treatment

Dietary treatment follows the guidelines for malnutrition [2, 28]. The dietitian will adjust the diet to ensure optimal intake, considering the client's preferences and usual eating habits. If normal food is insufficient to achieve the treatment goals, the dietitian will assess whether medical nutrition is needed and provide advice about it if necessary.

The following is recommended regarding protein intake per meal:

- consuming 20 grams of high-quality animal protein per meal, such as meat, fish, poultry, eggs, or dairy products; or
- consuming 25-30 grams of protein from average food sources, such as plant proteins (e.g., legumes, nuts, tofu) or a combination of animal and plant proteins.

The protein requirement may vary depending on the client's situation and health condition. For individuals with acute illnesses, a daily intake of 1.5-1.7 grams of protein per kilogram of body weight is recommended. For individuals with chronic diseases, a daily intake of 1.2-1.5 grams of protein per kilogram of body weight is recommended.

The following advice on protein and energy-enriched foods and snacks can be given to the client:

- Add protein-rich foods to meals and snacks, such as eggs, dairy products, lean meat, poultry, fish, tofu, legumes, and nuts.
- To increase energy intake, use full-fat dairy products, such as whole milk, yoghurt, and cheese, instead of low-fat.
- To increase energy density, add healthy fats to meals, such as avocado, nuts, seeds, olive oil, and fatty fish.
- Plan regular daily snacks to increase total calorie and protein intake.
- Choose energy-dense and protein-rich snacks, such as nuts, peanut butter on whole-grain crackers, yoghurt with fruit and granola, hummus with vegetables, cheese with whole-grain crackers, and energy bars with nuts and seeds.

4.1.3 Guideline for physical and functional training

For physical and functional training, a multi-component exercise program is recommended [29]. Practical components of this program include strength training, balance training, improving joint mobility, promoting functional mobility, endurance training, and exercises designed for daily activities. The recommended exercise frequency is two or three times a

week, lasting 30 to 60 minutes per session. The client can also do exercises independently without direct supervision from a professional.

Guidelines for the intensity of training:

- For progressive resistance training of the major muscle groups, a gradual increase in intensity is recommended, from 40% to 80% of the 1 repetition maximum. It is assumed that an intensity of 60 to 80% of 1 repetition maximum corresponds to an intensity of 8-12 repetition maximum [30]. Aim for 1 to 4 sets of 8-15 repetitions, depending on individual capacity and training goals [31].
- Aerobic training based on physiological training principles, aiming for an exertion level of 80% of the Heart Rate Reserve (HRR) or a BORG scale between 12-16.
- Gradually increase the difficulty of balance and ADL exercises.

The training program is individualised based on the client's capabilities and needs. It should aim to reach the desired intensity, with progress closely monitored and evaluated. In clients with malnutrition, both progressive resistance and aerobic training remain feasible, but should typically start at a lower intensity. The intensity should be gradually increased, considering the individual's nutritional status, tolerance, and functional capacity [28, 29].

4.2 Evaluation of treatment

The team should schedule regular evaluation moments to assess the client's progress. Recommended assessments should be used to obtain objective measurements of the severity and consequences of malnutrition and/or sarcopenia, as well as to evaluate the effectiveness of interventions aimed at addressing these conditions. If necessary, the treatment plan should be adjusted based on the evaluation. The treatment should be evaluated after three months to determine whether it should be continued or discontinued. Improvements in muscle strength and energy intake can occur in older adults after a period of two to three months [6, 32].

4.2.1 Recommended assessments and measurements for malnutrition

Recommended assessments for evaluating malnutrition include measurements of body composition and food intake, if the necessary measuring instruments are available [33].

Body composition

- BIA measurement (bioelectrical impedance analysis)

Food intake

- Dietary history method or a 24-hour recall
- Food diary
- Food frequency questionnaire

4.2.2 Recommended assessments for sarcopenia

Assessments for sarcopenia include measurements of muscle quantity and strength to determine the presence of sarcopenia, provided the necessary measuring instruments are available [11]. Measurements of physical functioning help assess the severity of sarcopenia. In addition to these assessments, balance, flexibility, and endurance measurements can be considered when the treatment also focuses on these outcomes.

Muscle quantity:

- BIA measurement (bioelectrical impedance analysis)

- Ultrasound

Muscle Strength:

- Handgrip strength
- Chair-stand test

Physical Functioning:

- Timed up-and-go test
- Short Physical Performance Battery (SPPB)
- 3/4/10 meter walk test

Follow-up data should be accurately recorded in the Electronic Patient Record (EPR) and communicated within the team. If the client does not make the expected progress, it is essential to identify the reasons and adjust the treatment plan.

4.3 Monitoring and follow-up

When the treatment for malnutrition and/or sarcopenia is completed, it remains essential for a professional from the team to continue monitoring the patient for possible relapse. The team should make work agreements for regular screening for (risk of) malnutrition and/or sarcopenia.

5. References

1. Wang DX, Yao J, Zirek Y, Reijnierse EM, Maier AB. Muscle mass, strength, and physical performance predicting activities of daily living: a meta-analysis. Journal of cachexia, sarcopenia and muscle. 2020.
2. Kruizenga H, Beijer S, Huisman-de Waal G, Jonkers-Schuitema C, Klos M, Remijnse-Meester W, Thijs A, Tieland BWM. Richtlijn ondervoeding. Stuurgroep ondervoeding. 2019.
3. Mayhew AJ, Amog K, Phillips S, Parise G, McNicholas PD, De Souza RJ, Thabane L, Raina P. The prevalence of sarcopenia in community-dwelling older adults, an exploration of differences between studies and within definitions: a systematic review and meta-analyses. Age Ageing. 2019.
4. Centraal Bureau voor de Statistiek. Gezonde levensverwachting; vanaf 1981. In: https://opendata.cbs.nl/statline/#/CBS/nl/dataset/71950ned/table?ts=1536152065267. 2024. Accessed 5 April 2024.
5. Wu P, Huang K, Chen K, Chou C, Tu Y. Exercise, nutrition, and combined exercise and nutrition in older adults with sarcopenia: a systematic review and network meta-analysis. Maturitas. 2021.
6. van Dongen EJ, Haveman-Nies A, Doets EL, Dorhout BG, de Groot LC. Effectiveness of a diet and resistance exercise intervention on muscle health in older adults: ProMuscle in practice. J Am Med Dir Assoc. 2020; doi:10.1016/j.jamda.2020.01.098.
7. Kaiser S, Patras J, Martinussen M. Linking interprofessional work to outcomes for employees: A meta-analysis. Res Nurs Health. 2018.
8. Pascucci D, Sassano M, Nurchis MC, Cicconi M, Acampora A, Park D, Morano C, Damiani G. Impact of interprofessional collaboration on chronic disease management: findings from a systematic review of clinical trial and meta-analysis. Health Policy. 2021.
9. Rawlinson C, Carron T, Cohidon C, Arditi C, Hong QN, Pluye P, Peytremann-Bridevaux I, Gilles I. An overview of reviews on interprofessional collaboration in primary care: barriers and facilitators. International Journal of Integrated Care. 2021.
10. Cederholm T, Barazzoni R, Austin P, Ballmer P, Biolo G, Bischoff SC, Compher C, Correia I, Higashiguchi T, Holst M. ESPEN guidelines on definitions and terminology of clinical nutrition. Clinical Nutrition. 2017.
11. Cruz-Jentoft AJ, Bahat G, Bauer J, Boirie Y, Bruyère O, Cederholm T, Cooper C, Landi F, Rolland Y, Sayer AA. Sarcopenia: revised European consensus on definition and diagnosis. Age Ageing. 2019.
12. Reeves S, Lewin S, Espin S, Zwarenstein M. Interprofessional teamwork for health and social care: 1st ed. Somerset: Blackwell Pub; 2010.
13. Barr H, Koppel I, Reeves S, Hammick M, Freeth D. Effective Interprofessional Education: Argument, Assumption and Evidence: Oxford: Wiley-Blackwell; 2006.
14. Gilbert JH, Yan J, Hoffman SJ. A WHO report: framework for action on interprofessional education and collaborative practice. J Allied Health. 2010.
15. Tsakitzidis G, Van Royen P. Leren interprofessioneel samenwerken in de gezondheidszorg: Antwerp: De Boeck; 2018.
16. van Dongen J, Goossens W, Spaans L, Beurskens S, van Bokhoven L. Doelgericht samenwerken in een MDO: vanzelfsprekend, of toch niet? De QuickScan Interprofessionele Team Samenwerking. Bijblijven. 2019.
17. Arnstein SR. A ladder of citizen participation. J Am Inst Plann. 1969.
18. Hart R. Children's Participation: From Tokenism To Citizenship. Innocenti Essays. 1992.
19. Sealy MJ, Haß U, Ottery FD, van der Schans CP, Roodenburg JLN, Jager-Wittenaar H. Translation and Cultural Adaptation of the Scored Patient-Generated Subjective Global Assessment: An Interdisciplinary Nutritional Instrument Appropriate for Dutch Cancer Patients. Cancer Nurs. 2018; doi:10.1097/NCC.0000000000000505.
20. Ottery FD. Definition of standardized nutritional assessment and interventional pathways in oncology. Nutrition. 1996.

21. Wijnhoven HA, Schilp J, de Vet HC, Kruizenga HM, Deeg DJ, Ferrucci L, Visser M. Development and validation of criteria for determining undernutrition in community-dwelling older men and women: The Short Nutritional Assessment Questionnaire 65. Clinical nutrition. 2012.
22. Voelker SN, Michalopoulos N, Maier AB, Reijnierse EM. Reliability and concurrent validity of the SARC-F and its modified versions: a systematic review and meta-analysis. Journal of the American Medical Directors Association. 2021.
23. Bahat G, Erdoğan T, İlhan B. SARC-F and other screening tests for sarcopenia. Current Opinion in Clinical Nutrition & Metabolic Care. 2022.
24. Brink E, van Rossum C, Postma-Smeets A, Stafleu A, Wolvers D, van Dooren C, Toxopeus I, Buurma-Rethans E, Geurts M, Ocké M. Development of healthy and sustainable food-based dietary guidelines for the Netherlands. Public Health Nutr. 2019.
25. Gezondheidsraad. Beweegrichtlijnen 2017. 2017.
26. Bull FC, Al-Ansari SS, Biddle S, Borodulin K, Buman MP, Cardon G, Carty C, Chaput J, Chastin S, Chou R. World Health Organization 2020 guidelines on physical activity and sedentary behaviour. Br J Sports Med. 2020.
27. Nederlandse Vereniging van Diëtisten (NVD), Koninklijk Nederlands Genootschap voor Fysiotherapie (KNGF), Ergotherapie Nederland (EN), Nederlandse Vereniging voor Logopedie en Foniatrie (NVLF), Beroepsvereniging Oefentherapie Cesar en Mensendieck (VvOCM), en Nederlandse Vereniging van Podotherapeuten (NVvP). Paramedische richtlijn kwetsbare ouderen: Praktijkrichtlijn. 2024.
28. Volkert D, Beck AM, Cederholm T, Cruz-Jentoft A, Goisser S, Hooper L, Kiesswetter E, Maggio M, Raynaud-Simon A, Sieber CC. ESPEN guideline on clinical nutrition and hydration in geriatrics. Clinical nutrition. 2019.
29. van Abbema R, de Vries NM, Weening-Dijksterhuis B, de Greef M, Hobbelen H. KNGF-standaard: Beweeginterventie kwetsbare ouderen. 2015.
30. Kraemer WJ, Adams K, Cafarelli E, Dudley GA, Dooly C, Feigenbaum MS, Fleck SJ, Franklin B, Fry AC, Hoffman JR. American College of Sports Medicine position stand. Progression models in resistance training for healthy adults. Med Sci Sports Exerc. 2002.
31. Belgian Society for GERONTOLOGY and GERIATRICS. SARCOPENIA GUIDELINE. In: https://geriatrie.be/the-bsgg/initiatives/works-and-contributions/sarcopenia-guidelines/. 2020. Accessed 29-05 2024.
32. Reinders I, Volkert D, de Groot LC, Beck AM, Feldblum I, Jobse I, Neelemaat F, de van der Schueren MA, Shahar DR, Smeets ET. Effectiveness of nutritional interventions in older adults at risk of malnutrition across different health care settings: Pooled analyses of individual participant data from nine randomized controlled trials. Clinical nutrition. 2019.
33. Kruizenga H, Beijer S, Huisman-de Waal G, Jonkers-Schuitema C, Klos M, Remijnse-Meester W, Thijs A, Tieland BWM. Richtlijn ondervoeding. Stuurgroep ondervoeding. 2017.
34. Ter Beek L, Banning LB, Visser L, Roodenburg JL, Krijnen WP, van der Schans CP, Pol RA, Jager-Wittenaar H. Risk for malnutrition in patients prior to vascular surgery. The American Journal of Surgery. 2018.
35. Banning LB, Ter Beek L, El Moumni M, Visser L, Zeebregts CJ, Jager-Wittenaar H, Pol RA. Vascular surgery patients at risk for malnutrition are at an increased risk of developing postoperative complications. Ann Vasc Surg. 2020.
36. FD Ottery. Scored Patient-Generated Subjective Global Patient Identification Information Assessment (PG-SGA). In: https://pt-global.org/wp-content/uploads/2021/02/PG-SGA-version-4.3.20-std-logo-non-metric.pdf. 2020. Accessed 30-06 2025.
37. Dutch Malnutrition Steering Group. SNAQ 65+. In: https://www.kenniscentrumondervoeding.nl/wp-content/uploads/2024/01/Snaq_65__engels_final-1.pdf. Accessed 30-06 2025.

38. Cederholm T, Jensen GL, Correia M, Gonzalez MC, Fukushima R, Higashiguchi T, Baptista G, Barazzoni R, Blaauw R, Coats A. GLIM criteria for the diagnosis of malnutrition–a consensus report from the global clinical nutrition community. Journal of cachexia, sarcopenia and muscle. 2019.
39. Gascón-Ruiz M, Casas-Deza D, Torres-Ramón I, Zapata-García M, Alonso N, Sesma A, Lambea J, Álvarez-Alejandro M, Quílez E, Isla D. Comparation of different malnutrition screening tools according to GLIM criteria in cancer outpatients. Eur J Clin Nutr. 2022.
40. Meetinstrumenten in de zorg. Uitgebreide toelichting van het meetinstrument Timed Chair-Stand-Test (TCST/TCS) Overkoepelende naam: Chair Stand Test (CST). In: https://meetinstrumentenzorg.nl/instrumenten/timed-chair-stand-test/. 2023. Accessed 05-05 2024.
41. Langius J, Visser W, Kruizenga H, Reijven N. Meetprotocol handknijpkracht.
42. Roberts HC, Denison HJ, Martin HJ, Patel HP, Syddall H, Cooper C, Sayer AA. A review of the measurement of grip strength in clinical and epidemiological studies: towards a standardised approach. Age Ageing. 2011.
43. Heidi Zweers, Julia Korzilius, Hinke Kruizenga, Wesley Visser, Inez Jans, en Anneke Droop. Single Frequency Bio-Impedantie Analyse. In: https://nutritionalassessment.nl/wp-content/uploads/2024/01/NAP-SF-BIA-SOP-versie-7.pdf. 2023. Accessed 05-05 2024.
44. Inez Jans. Meetprotocol MF-BIA InBody S10. In: https://nutritionalassessment.nl/wp-content/uploads/2024/01/SOP-MF-BIA-InBody-S10-versie-1-12-2021.pdf. 2021. Accessed 05-05 2024.
45. Earthman CP. Body composition tools for assessment of adult malnutrition at the bedside: a tutorial on research considerations and clinical applications. J Parenter Enteral Nutr. 2015.
46. Sergi G, De Rui M, Veronese N, Bolzetta F, Berton L, Carraro S, Bano G, Coin A, Manzato E, Perissinotto E. Assessing appendicular skeletal muscle mass with bioelectrical impedance analysis in free-living Caucasian older adults. Clinical nutrition. 2015.
47. Dodds RM, Syddall HE, Cooper R, Benzeval M, Deary IJ, Dennison EM, Der G, Gale CR, Inskip HM, Jagger C. Grip strength across the life course: normative data from twelve British studies. PloS one. 2014.
48. Cesari M, Kritchevsky SB, Newman AB, Simonsick EM, Harris TB, Penninx BW, Brach JS, Tylavsky FA, Satterfield S, Bauer DC. Added value of physical performance measures in predicting adverse health-related events: results from the Health, Aging and Body Composition Study. J Am Geriatr Soc. 2009.
49. Studenski SA, Peters KW, Alley DE, Cawthon PM, McLean RR, Harris TB, Ferrucci L, Guralnik JM, Fragala MS, Kenny AM. The FNIH sarcopenia project: rationale, study description, conference recommendations, and final estimates. Journals of Gerontology Series A: Biomedical Sciences and Medical Sciences. 2014.
50. Gould H, Brennan SL, Kotowicz MA, Nicholson GC, Pasco JA. Total and appendicular lean mass reference ranges for Australian men and women: the Geelong osteoporosis study. Calcif Tissue Int. 2014.
51. Goossens W, van Dongen JvD. Interprofessionele teams in hun kracht: Neer: Kloosterhof BV; 2024.

Appendix 1 Team evaluation with the QuickScan interprofessional team collaboration (translated from Dutch)

The QuickScan can be used as a self-assessment tool to evaluate the quality of collaboration within the team and to gather input for reflection and subsequent optimisation [16].

In the QuickScan, team members assess statements based on seven categories on a scale from 0 (not present) to 10 (excellent). Each team member is also asked to indicate whether each statement is an important development point for the team or network. The scan concludes with three open questions where additional explanations can be provided. Completing the scan takes approximately 15 minutes.

Instructions for completing the form

- **First**, indicate your opinion on each statement by providing a rating on a scale from 1 to 10.
  A score of **1** means: *Not present* (a gap, missing element, or lacking attention)
  A score of **10** means: *Excellent presence* (best practice, exemplary, publication-worthy)
  You may choose any number between 1 and 10.
- **Second**, indicate whether you consider this item a development point for your team.
  Yes = This is a development point for our team.
  No = This is currently not a development point for our team.
- **Finally**, a few open-ended questions will follow regarding collaboration within your team or network.

QuickScan Interprofessional Team Collaboration [De QuickScan Interprofessionele Team Samenwerking] [16] (translated from Dutch)		
**Statements on Interprofessional Team Collaboration per theme**	**Assessment**	**Development point**
	**Rating 1 to 10**	**Yes/no**
**Theme 1<br>Context awareness**		
1. We are aware of trends, national developments, and policies (laws and regulations) that affect collaboration within our team.		
2. We are aware of the influence of cultural aspects on our collaboration.		
3. We understand the organisational background of our team members (e.g. the mission and interests of their parent organisations).		
4. We have a clear overview of our formal and informal network.		
5. We know each other's professional expertise.		

6. We are familiar with each other's personal strengths.		
**Theme 2**<br>**Shared values**		
7. As a team, we are aware of each member's personal core values.		
8. As a team, we have insight into our shared values.		
9. Our (parent) organisations support us in working according to these shared values.		
10. We encourage one another to work in line with our shared values.		
11. We have translated our shared values into a team mission, vision, and goals.		
12. We have translated our shared values into collective ambitions.		
**Theme 3**<br>**Leadership**		
13. As team members, we take personal responsibility to support our team's development (= personal leadership).		
14. We regard leadership as a shared responsibility of the entire team (= shared leadership).		
15. We have leadership in our team that inspires us to achieve our shared goals.		
16. We apply different leadership styles depending on the situation (= situational leadership).		
17. Our team leadership encourages us to bring out the best in ourselves.		
18. Our leadership contributes to a sense of psychological safety within the team.		
**Theme 4**<br>**Structure en organisation**		
19. We have clarity about the frameworks and boundaries within which we operate as a team.		
20. We have a clear and defined team assignment and team goals.		
21. Our team has an appropriate size and composition.		
22. We have a clear division of roles (e.g. chairperson, minute-taker, case presenter), which makes tasks and responsibilities clear.		
23. We operate within a clear structure, including division of tasks and roles, an agenda, working methods, and meeting frequency.		
24. We employ a shared working method or framework for collaboratively discussing cases.		
**Theme 5**		

**Group dynamics and interaction**		
25. We have a positive group climate (mutual trust, team spirit).		
26. Each participant is acknowledged for their presence, contributions, and personal functioning.		
27. We consult and question each other in an open and unbiased manner.		
28. We complement one another and make use of this diversity (= complementarity).		
29. We can communicate openly about our interpersonal relationships.		
30. During meetings, speaking time is well balanced among all team members.		
**Theme 6**<br>**Psychological safety**		
31. In our team, we feel safe to acknowledge and discuss mistakes.		
32. We foster an open atmosphere in which feedback, differences of opinion, tension, and conflict can be addressed constructively.		
33. We can discuss negative experiences or emotions in a constructive manner.		
34. We trust in each other’s good intentions.		
35. We address one another directly and give constructive feedback and/or feedforward.		
36. We investigate signs of unsafety in the team and do not avoid these conversations.		
**Theme 7**<br>**Learning capacity**		
37. We learn from and with one another, regularly exchange knowledge and experience, and translate this into our practice.		
38. We have a team culture in which learning, making mistakes, and curiosity are accepted as normal.		
39. We invest in the development of (new) team members, aimed at strengthening the team as a whole.		
40. We take advantage of the opportunities provided by our organisations to foster learning.		
41. As a team, we respond proactively to external developments and influences.		
42. We have embedded joint reflection and monitoring into our team's routines (e.g., on team development, effectiveness, and composition).		
**Theme 8**<br>**Entrepreneurship**		

43. Our team has well-organised business operations (financial, organisational, human resources, and materials).		
44. We use systems that support our work and improve efficiency.		
45. We communicate externally what makes our team unique and effective.		
46. We pay attention to public relations and communication as a team.		
47. As a team, we have the courage to be innovative.		
48. We are market-oriented and anticipate trends and changes in our environment.		

Open-ended questions

After completing the QuickScan, we invite you to reflect on and draw conclusions about the collaboration within your team or network.

What are we good at as a team, and what do I want to preserve?

What is the most important thing I would like to change in the team?

What actions do I believe our team should take?

Which actions within our team will I take ownership of?

Note: This is an unofficial English translation of the Quickscan IPT, originally developed by van Dongen, J., & Goossens, W. (2022). QUICKSCAN IPT Voor reflectie op de samenwerking in een interprofessioneel team [Quickscan IPT: For reflection on interprofessional team collaboration] (Version 16-09-2022) [Dutch; unpublished instrument]. Zuyd University of Applied Sciences.

Used with permission from the original authors. Not for redistribution without their consent.

Appendix 2 Detection guide for older adults, caregivers, and the social network

Nutrient deficiencies and unintentional weight loss are common among people aged 65 years and older

How to recognise nutrient deficiencies and weight loss?

- Your clothes, belt, or watch fit more loosely
- Wounds heal slowly
- You often feel tired
- You have difficulty shopping and cooking

Unintentional weight loss is more common:

- When having problems with chewing or swallowing
- During illnesses that increase the body's energy requirements
- When having stomach or intestinal problems
- When eating less than before

Do you suspect nutrient deficiencies or unintentional weight loss in yourself?

Discuss your suspicions with your healthcare provider, such as your general practitioner, dietitian, district nurse, or geriatric specialist. Together, you can determine if any further action is needed.

This detection guide (v.1.1) has been developed for people aged 65 and older and their social network, as part of an interprofessional care pathway for malnutrition.

Backside detection guide

Loss of muscle strength is common among people aged 65 years and older and is not a normal consequence of ageing

InterGAIN

How to notice that your muscles are less strong?

- Difficulty getting up from a chair
- Difficulty climbing stairs
- Walking more slowly or unsteadily
- You fall more frequently
- You become tired or short of breath more quickly

Loss of muscle strength occurs more frequently:

- When having lack of physical activity
- In people who are malnourished
- During and after illness

Do you suspect loss of muscle strength in yourself?

Discuss your suspicions with your healthcare provider, such as your general practitioner, physiotherapist, district nurse, dietitian, or specialist. Together, you can determine if any further action is needed.

This detection guide (v.1.1) has been developed for people aged 65 and older and their social network, as part of an interprofessional care pathway for malnutrition.

Appendix 3 Detection guide for professionals

Detection guide
Warning signs for (risk of) malnutrition and sarcopenia
For health- and social care professionals

Malnutrition is an acute or chronic condition in which a deficiency or imbalance of energy, protein, and other nutrients results in measurable adverse effects on body composition, function, and clinical outcomes.

Sarcopenia is a skeletal muscle disorder characterised by progressive and systemic loss of skeletal muscle mass and strength, with a risk of adverse outcomes such as physical limitations, reduced quality of life, and mortality.

Risk factors for malnutrition
- Gastrointestinal problems
- Conditions resulting in increased energy requirements
- Polypharmacy
- Chewing and swallowing difficulties
- Reduced appetite
- Changes in taste and smell

Risk factors for sarcopenia
- Malnutrition
- Low body mass index (BMI)
- Lack of physical activity
- Older age
- Smoking

Characteristics and consequences of malnutrition
- Unintentional weight loss
- Loss of muscle mass
- Delayed recovery after illness or injury
- Reduced energy and unexplained fatigue
- Difficulty or reluctance to shop and/or cook independently
- Complains of feeling cold quickly
- Fall incidents

Characteristics and consequences of sarcopenia
- Muscle weakness
- Reduced muscle function
- Decreased endurance
- Loss of muscle mass

See the reverse side for actions to take when you recognise signs of malnutrition and/or sarcopenia in your client

This detection guide has been developed as part of the interprofessional care pathway for malnutrition and sarcopenia in community-dwelling older adults.

Backside detection guide

When you recognise these signs, it is important to discuss them with your client and/or their social network.

↓

If malnutrition and/or sarcopenia is suspected, it is important to screen. If you are not skilled in screening or do not have the appropriate tools, proceed to the next step.

- Use the PG-SGA Short Form or the SNAQ 65+ to screen for malnutrition.
- Use the handgrip strength test and chair stand test to screen for sarcopenia.

The implementation and normative values of the screening instruments are described in the interprofessional care pathway for (risk of) malnutrition and sarcopenia in community-dwelling older adults.

↓

Contact the dietitian and physiotherapist in case of (risk of) malnutrition and/or sarcopenia, and in consultation with the client and/or their social network

↓

The dietitian and physiotherapist will initiate dietary treatment, muscle, and functional training upon a positive screening result.

Appendix 4 Screening and diagnosing malnutrition

PG-SGA Short Form

The most recent version of the Patient-Generated Subjective Global Assessment (PG-SGA) can be downloaded from https://www.pt-global.org/. Sections 1 to 4 of the PG-SGA are called the PG-SGA Short Form (PG-SGA SF). The results of the PG-SGA SF can fall into one of the following three risk groups: 0-3 points = low risk; 4-8 points = moderate risk; 9 points or more = high risk [34, 35].

Scored Patient-Generated Subjective Global Assessment (PG-SGA)

History: Boxes 1 - 4 are designed to be completed by the patient.
[Boxes 1-4 are referred to as the PG-SGA Short Form (SF)]

Patient Identification Information

1. Weight *(See Worksheet 1)*

In summary of my current and recent weight:

I currently weigh about ____kg
I am about _____ cm tall

One month ago I weighed about _____ kg
Six months ago I weighed about _____ kg

During the past two weeks my weight has:
☐ decreased (1) ☐ not changed (0) ☐ increased (0)

Box 1 ☐

2. Food intake: As compared to my normal intake, I would rate my food intake during the past month as
- ☐ unchanged (0)
- ☐ more than usual (0)
- ☐ less than usual (1)

I am now taking
- ☐ *normal food* but less than normal amount (1)
- ☐ little solid food (2)
- ☐ only liquids (3)
- ☐ only nutritional supplements (3)
- ☐ very little of anything (4)
- ☐ only tube feedings or only nutrition by vein (0)

Box 2 ☐

3. Symptoms: I have had the following problems that have kept me from eating enough during the past two weeks (check all that apply)
- ☐ no problems eating (0)
- ☐ no appetite, just did not feel like eating (3)
- ☐ vomiting (3)
- ☐ nausea (1)
- ☐ diarrhea (3)
- ☐ constipation (1)
- ☐ dry mouth (1)
- ☐ mouth sores (2)
- ☐ smells bother me (1)
- ☐ things taste funny or have no taste (1)
- ☐ feel full quickly (1)
- ☐ problems swallowing (2)
- ☐ fatigue (1)
- ☐ pain; where? (3) ________________
- ☐ other (1)** __________________

**Examples: depression, money, or dental problems

Box 3 ☐

4. Activities and Function:
Over the past month, I would generally rate my activity as:
- ☐ normal with no limitations (0)
- ☐ not my normal self, but able to be up and about with fairly normal activities (1)
- ☐ not feeling up to most things, but in bed or chair less than half the day (2)
- ☐ able to do little activity and spend most of the day in bed or chair (3)
- ☐ pretty much bed ridden, rarely out of bed (3)

Box 4 ☐

The remainder of this form is to be completed by your doctor, nurse, dietitian, or therapist. Thank you.

Additive Score of Boxes 1-4 ☐ **A**

©FD Ottery 2005, 2006, 2015 v3.22.15
email: faithotterymdphd@aol.com or info@pt-global.org

Figure 3.1 PG-SGA Short Form [36]

SNAQ 65+

The SNAQ65+ helps guide treatment decisions by categorizing older adults into three groups: (1) undernutrition, (2) at risk of undernutrition, and (3) no undernutrition [21]. The full SNAQ65+ tool is available at www.fightmalnutrition.eu.

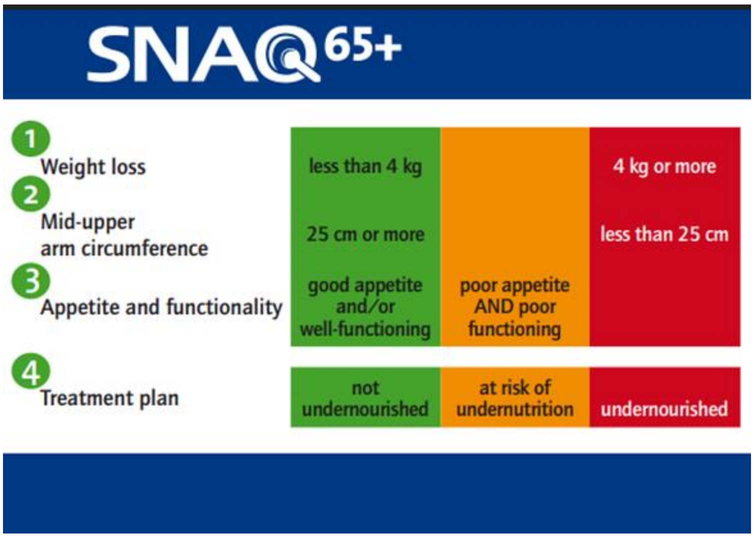

Figure 3.2 Items and risk classification of the SNAQ 65+ tool [37]

GLIM-criteria

Malnutrition is present when a patient meets at least one phenotypic (characteristic) factor AND at least one etiologic (causal) factor, as indicated in the table below [38]. Severe malnutrition is determined when a patient meets at least one phenotypic (characteristic) factor for severe malnutrition. The procedure for bioelectrical impedance analysis (BIA) is described in Appendix 4.

Table 3.1 GLIM criteria for malnutrition [39]

	**Etiologic Criteria**		**Phenotypic Criteria**		
	**Reduce food intake**	**Inflammation**	**Involuntary weight loss (%)**	**Low body mass index**	**Reduced muscle mass**
**Stage 1** Moderate malnutrition (Requires 1 phenotypic criterion that meets this grade)	≤50% of complete ration during >1 week or any reduction during >2 weeks<br><br>OR<br><br>Any chronic gastrointestinal condition that adversely impacts food assimilation or absorption	Acute disease or chronic inflammatory disease	5–10% within the last 6 months<br>or<br>10–20% beyond 6 months	<20 if <70 years <22 if ≥70 years	Mild to moderate deficit (per validated assessment methods)
**Stage 2** Severe malnutrition (Requires 1 phenotypic criterion that meets this grade)			>10% within the last 6 months<br>or<br>>20% beyond 6 months	<18.5 if <70 years<br><br><20 if ≥70 years	Severe deficit (per validated assessment methods)

Appendix 5 Screening and diagnosing sarcopenia

EWGSOP2-criteria

The European Working Group on Sarcopenia in Older People (EWGSOP2) criteria for diagnosing sarcopenia include measuring muscle strength, such as handgrip strength and the chair stand test, as well as evaluating muscle quantity or quality using methods like DEXA, BIA, CT, or MRI scans [11]. BIA is often used in practice due to the accessibility, affordability, and portability of the equipment. Low muscle strength is an indication of possible sarcopenia and justifies further investigation and the initiation of treatments. By also assessing muscle quantity or quality, sarcopenia can then be confirmed.

Chair stand test

The client is asked to stand up from the chair and sit down again five times as quickly as possible without using their arms. Use a sturdy chair; preferably, place the backrest against the wall for safety. The client's starting position is sitting on a chair with arms crossed over the chest. The time in seconds is recorded [40]. The test takes approximately 30 seconds.

Handgrip strength

Measure according to Robert et al. as described in the Standard Operating Procedure (SOP) Handgrip Strength [41, 42]. The client sits relaxed, upright, with the forearms at a 90° angle and the wrist in a neutral position (thumb pointing up). Adjust the grip of the handgrip dynamometer so that it fits comfortably in the participant's hand, with the middle phalanx of the middle finger forming a 90° angle around the grip. Encourage the participant to squeeze as hard as possible until the "peak-hold" needle stops rising. Allow 30 seconds of rest between each measurement. Perform at least two, preferably three measurements per hand. The test takes approximately 5 minutes.

Bioelectrical impedance analysis (BIA)

Follow the Standard Operating Procedure (SOP) SF-BIA on the Nutritional Assessment Platform (NAP) website [43, 44]. When performing bioelectrical impedance analysis (BIA), it is preferred to use a method where the person is lying down, as this ensures an even distribution of fluid in the body. When measured standing, the electrodes under the feet can be less reliable due to variations in skin thickness caused by callus formation [45]. Appendicular skeletal muscle mass (ASM) is calculated using the formula by Sergi et al. [46] in accordance with the GLIM and EWGSOP2 criteria. As described in the SOP SF-BIA, specific formulas are available for Asian people and people with COPD. The calculation sheet on https://zakboekdietetiek.nl/uitslag-impedantie-volwassenen/ can be used. The BIA measurement typically takes about 15 minutes.

Formula Sergi et al. (Kaukasian) [46]:

-3,964 + (0,227 * RI) + (0,095 * weight) + (1,384 * gender) + (0,064 * Xc)

Table 4.1 Cut-off values for low muscle mass and quality

**Test**	**Cut-off values men**	**Cut-off values Women**
EWGSOP2 sarcopenia cut-off values low muscle strength		
Handgrip strength **[47]**	**<27kg**	**<16kg**
Chair stand test **[48]**	**>15s for 5 times sit to stand**	
EWGSOP2 sarcopenia cut-off values low muscle quantity		
ASM **[49]**	**<20 kg**	**<15 kg**
ASM/(length)^2 **[50]**	**<7.0 kg/m^2**	**<5.5 kg/m^2**

ASM = Appendicular skeletal muscle mass

Appendix 5 Guidelines for team agreements

1. **Screening for malnutrition and sarcopenia**
   Use validated screening tools:
    - Malnutrition: Use the [PG-SGA Short Form / SNAQ65+].
    - Sarcopenia: Use the handgrip strength and chair stand test.
2. **Client meetings**
    - Location: [physical/digital]. Note the address and/or communication platform.
    - Frequency [e.g., weekly/bi-weekly/monthly].
    - Day/Time: [e.g., Monday at 10:00 am, every 4 or 6 weeks].
    - Registration: Clients are registered via [platform/person] with a completed Client Meeting Form.
    - Reporting: Reports are prepared by [specific person or role], and all agreements are documented in the EHR by [specific person or role].
    - Feedback: Outcomes are discussed with the client by the designated contact person of the client.
3. **Communication lines**
    - Internal communication via [e.g., regular meetings, emails, communication apps].
    - External communication via [e.g., phone, email].
4. **Use of Electronic Health Records (EHR)**
    - Uniform use by [e.g., standard templates for notes, consistent use of abbreviations and terminology].
5. **Information management and sharing**
    - Information is shared via [e.g., shared online documents, EHR notes, secure communication channels].
    - All information is handled according to applicable privacy and security guidelines.

Appendix 6 Guidelines for documenting team agreements per client

1. Client care coordinator

- The client care coordinator is [name of the person].
- Responsibilities
  - Monitoring the client's progress
  - Acting as the primary contact point for team members
  - Contact person for the client, caregiver, and their family

2. Involved professionals

Note the names, roles, specialisations, and contact details of the involved professionals

Name	Roles and specialisations	Contact details

3. Treatment goals

- Personal goals and wishes of the client
- Short- and long-term goals of the interprofessional treatment
- Evaluation of progress towards these goals

4. Interventions and coordination

- Brief description of interventions
- Main person responsible for each intervention

5. Task coordination and redistribution

- List of all tasks to be performed for the client's care
- Clear division of tasks within the team
- Description of which professional takes on which task

Appendix 7 Guidelines for client meetings

Guidelines for client discussions

- Client discussions are not optional; each involved discipline should be present
- Client input is standardised, for example, via the client meeting form
- A chairperson and minute-taker are appointed:

Chairperson

- Coordination
- Managing the agenda
- Summarising
- Initiating reflection
- Keeping track of the schedule
- Facilitating equal input

Minute-taker

- Taking minutes during the meeting
- Documenting agreements (see table of documented agreements during client meeting)

- Everyone is informed about the content of the discussion
- Each participant prepares for the discussion
- There is a fixed meeting structure

Meeting structure agenda (example)

- Start
- Announcements
- Fixed part (3-4 clients according to schedule)
- Variable part (urgent cases)
- Wrap up

- Each participant is responsible for ensuring that the adjusted care/treatments are recorded in the electronic health record (EHR)
- The client's contact person is responsible for informing the client and, if applicable, their network about the outcome of the client discussion

Use the client discussion step-by-step plan to conduct the meeting purposefully based on concrete questions.

Step-by-step plan for client discussions

The step-by-step plan for client discussions consists of various phases that ensure the client's perspective is central and that the discussion is goal-oriented and based on a concrete question [51]. Here is an overview of the plan:

1	2	3	4	5	6
**Description of the client's situation**	**Goals and motivation**	**Analysis**	**Inventory of possible action proposals**	**Formulate concrete agreements**	**Evaluation**
What is the situation of the client (and their relatives)?<br>What is the question for this meeting?<br>What is the reason for bringing this up?<br>What result do we want to achieve?	What are the client's goals and preferences?<br>What is the team's perspective on the client's goals?	What do the client and their environment need to achieve the goals?<br>What can the clients do themselves?<br>What can relatives contribute?<br>What are the possibilities from the current field?	What can we recommend to the client and their relatives to achieve the goal? (actions)<br>Which actions best match the client's preferences?	Which actions will the client and their relatives take?<br>Who can contribute to the implementation of possible actions?<br>What concrete (care) agreements do we make? Who takes on what?	Does the presenter have sufficient answers to their question?<br>Is monitoring and evaluation needed at a later time?

**Form Client discussion**	
Date	
Clients name	
Date of birth	
Involved parties	
Client’s main goal	
Concrete question and reason for bringing it up for client discussion	

Brief description of the client's situation	

Recorded agreements during client discussions			
**Number**	**Agreement**	**Responsible**	**Timeline**
**1**			
**2**			
**3**			
**4**			
**5**			
**6**			

Adapted and translated from:
Jerôme van Dongen & Wim Goossens (2024). *Interprofessionele teams in hun kracht*: Ontwikkelen, begeleiden & coachen [Interprofessional teams in their strength: Developing, guiding & coaching]. Neer: Kloosterhof Neer B.V. ISBN 978-90-79816-41-0. (Dutch)

Used with permission from the publisher. Copyright remains with the publisher.
